# Evaluating the impact of video cameras on participant behaviour in research: a systematic review and meta-analysis

**DOI:** 10.1186/s13643-025-03055-z

**Published:** 2026-01-24

**Authors:** Matheesha Herath, Scarlotte Kulas, Jessie Martin, Ellie C Treloar, Jesse D Ey, Emma L Bradshaw, Jarrod DeSilva-White, Suzanne Edwards, Martin Bruening, Guy J Maddern

**Affiliations:** 1 Department of Surgery, Adelaide University, The Queen Elizabeth Hospital, 28 Woodville Road, Woodville South, SA 5011 Australia; 2 School of Public Heath, Adelaide University, North Terrace, Adelaide, SA 5000 Australia

**Keywords:** Hawthorne effect, Video camera, Research methods, Behaviour

## Abstract

**Background:**

Human behavioural research is often clouded with the risk that study results may be contaminated by the participant’s awareness that they are being observed. Direct observation by a person is associated with this phenomenon, but limited data exists evaluating this Hawthorne Effect when less invasive video recording devices are used. Here we present the first quantitative analysis to identify the extent to which this occurs, based on self-reported behavioural change when cameras are used.

**Methods:**

Searches of MEDLINE, Embase, Emcare, PsycINFO, CINAHL, and Google Scholar were performed on 01/12/2022. No limitations were set. The primary outcome was the proportion of participants who changed their behaviour due to awareness of being recorded. Two blinded reviewers performed screening in accordance with PRISMA guidelines. *I*^2^ statistic was used to assess for heterogeneity and a random effects model was subsequently applied for the meta-analysis.

**Results:**

Preliminary searches identified 1728 publications. After screening, twenty-eight studies were included in the final analysis involving 2586 participants. Nine publications were suitable for quantitative analysis of the primary outcome. Pooled analysis using a random-effects model demonstrated the proportion of participants who reported behavioural change because of the camera was 15% (95% CI 0.08, 0.23) [*I*^2^ = 96.16%].

**Conclusion:**

The presence of a video camera may cause behavioural change in a small proportion of study participants. Cameras may cause a much lower rate of reactivity compared to a direct human observer. The heterogeneity and high risk of bias of the publications highlight the need for further high-quality research into this subject area.

**Systematic review registration:**

PROSPERO CRD42022370498

**Supplementary Information:**

The online version contains supplementary material available at 10.1186/s13643-025-03055-z.

## Introduction

The complexity of conducting human research can be as challenging as human nature itself. The crude adage of science that is echoed in classrooms, ‘that it is impossible to prove a hypothesis,’ underpins our academic foundation. Reduction of bias in research results in higher quality, more generalisable results [1]. This can be especially challenging in human observational research. Every experiment that involves direct or indirect observation is at risk of a bias known as ‘The Hawthorne Effect’. In a landmark study, workers were observed in different conditions in an attempt to determine variables that impacted productivity. Review of the data demonstrated that call cohorts being observed increased their output [2]. This phenomenon of behavioural change associated with awareness of observation was dubbed “The Hawthorne Effect” [2–7]. The impact of this effect has been debated in observational research and reports are heavily conflicting [5, 6, 8–11].

The importance of the Hawthorne effect has been debated with contradictory evidence reported; however, contemporary understanding is that it does exist, and it does impact studies, though the exact degree to which the Hawthorne Effect impacts study outcomes is incompletely understood [2, 5, 8, 10–12]. Studies where investigators are directly observing participants and physically visible to participants are at greater risk of this bias [13]. There is a paucity of evidence examining the impact of the Hawthorne Effect using video recording devices in place of direct human observation. In modern research, many studies are observational in nature, and therefore, cameras have become increasingly utilised in research. The degree to which observational research with video cameras is impacted by the Hawthorne effect is important because this ‘reactivity bias’ can have significant implications regarding how we interpret studies involving human behaviour.

Comparison studies attempt to minimise their risk by ensuring all cohorts are exposed to the same observational conditions [6, 11, 14]. Other studies suggest exploiting the phenomenon and recommend the presence of observers to influence a particular behaviour [15, 16]. The arguments against the existence of the Hawthorne Effect criticise the original experiments or call for further understanding of the complexity of human behaviour [6, 7, 10, 11]. The debate about the existence of the Hawthorne Effect suggests that it may have variable penetrance, but there could be methods to mitigate its impact.

Humans harbor a distrust toward surveillance, and we have an instinctive discomfort when we feel like we are being watched [17, 18]. Despite this, we have become accustomed and conditioned to the presence of cameras in our lives. Travelers passing through LAX airport will be in contact with several of the 3000 cameras that are permanent fixtures in one of the world’s busiest airports [19]. Over 6 billion people own smartphones around the world [20]. Cameras have become a part of life in 2023, and we are not just acclimated to them, but reliant on them. Without peer-to-peer video conferencing software, many industries would have ground to a halt, education courses would have ceased, and people would have struggled to maintain social connection during isolation [21]. The transition from video cameras initially being novel devices to mundane necessities in life has resulted in societal acceptance of these devices [22, 23]. Habituation effects occur with repeated exposure, and younger generations are demonstrating this as video-based education initiatives demonstrate reduced reactivity as they become more commonplace [23, 24].

The foundation of quality human behavioural research relies upon the validity of conclusions. As the world has evolved, so too has research. Technology has not only made our lives more convenient, but it has also enabled us to capture and process information in unprecedented ways. Machines allow us to remove emotion and human error from a situation. This quest for objectivity has seen human observational research transition from using persons with clipboards to discrete surveillance cameras. The implications of this strongly impact the generalisability of study findings. To date, there is no review examining the impact of video cameras on participant behaviour. The aim of this systematic review and meta-analysis is to provide quantitative analysis of the impact of the Hawthorne Effect in research using video recording devices. Secondary outcomes include an analysis of factors related to the conduct of observation research using video cameras to provide guidance to minimise or mitigate the Hawthorne effect in future studies.

## Methods

This systematic review and meta-analysis was prospectively registered on the PROSPERO database (CRD42022370498) and conducted in line with Meta-analysis of Observational Studies in Epidemiology (MOOSE) guidelines and PRISMA Guidelines (Appendix 1).

Comprehensive search strategy was developed by the first author and two senior reference librarians. Search was conducted using the terms listed in Appendix 2. No language, date, or publication type limitations were set. Search was conducted on 01/12/2022 across five biomedical databases: MEDLINE (including PubMed), Embase, Emcare, PsycINFO, and CINAHL. Manual search of Cochrane Library and trial registries was conducted.

Eligible articles for inclusion were those that included use of any form of video camera and reported behaviour change relative to the video camera. This included Randomised control trials, non-randomised control trials, and cohort studies. Conference abstracts, scientific posters, articles describing methodology without reported participant behaviour, or studies using direct human observation without the use of a camera were excluded (Appendix 3).

Screening of articles by abstract and full text was performed by two independent reviewers (MH, SK) with disagreements resolved by a 3rd independent reviewer (JM) in accordance with PRISMA guidelines using the Covidence web-based systematic review management software. References of included studies were interrogated to assess for further eligible studies.

Data extraction was conducted by two independent reviewers (JM,MH) and then validated by two other reviewers (ET, SK) in alignment with a pre-defined extraction plan (Appendix 4). Where data was missing, corresponding authors were contacted.

Primary outcomes included objective changes to task completion relative to the use of a camera, observable (e.g. looking at camera or hiding from camera) and self-reported measures of behavioural change related to camera presence (awareness of camera, feelings of concern or anxiety related to camera presence). Secondary outcomes included measures of camera use methodology such as positioning of camera, size of camera, time spent in front of camera. Additional data points collected included year and country of study, participant demographics.

### Risk of bias

For all included studies, the risk of bias was assessed using the Newcastle Ottawa Scale (NOS) by two independent reviewers (ET, JE) with disagreements resolved by a third (MH).

### Statistical analyses

Statistical analysis was conducted by an experienced biostatistician (SE). For primary outcomes, the I^2^ statistic was used to evaluate heterogeneity (with *I*^2^ > 50% indicating significant heterogeneity) as was Cochran’s *Q* *p* value (with *p* value < 0.05 indicating significant heterogeneity). A random-effects model was used throughout, and Forest Plots are provided. For secondary outcomes, descriptive statistics were used.

Data analyses were performed using Stata Statistical Software: Release 15.1 College Station, TX: StataCorp LP.

## Results

**Fig. 1:**
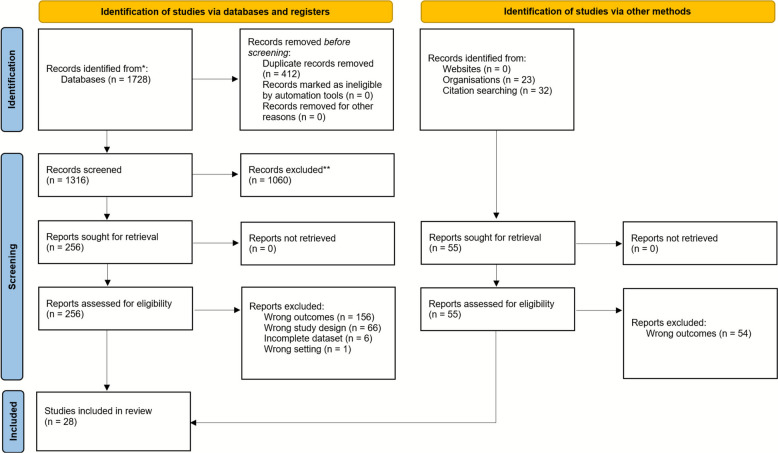
PRISMA flowchart

Preliminary search results yielded 1728 publications. After duplicate removal and filtration, 28 studies were included in the final analysis (Fig. 1). Twenty-two were observational studies, three were qualitative projects, two were non-randomised experimental studies, and one was a randomised controlled trial. These papers were published between 1984 and 2022 and involved 2586 participants. Ten publications reported baseline sex characteristics (*n* = 1070) with a female predominance of 63.6%. Sixteen papers reported behaviour changes in response to the presence of a video camera as the primary outcome; the remainder were secondary outcomes [25–52]. There was considerable heterogeneity in the reporting of results. In crude comparison on a binary scale of whether cameras caused behavioural change, 23 publications reported that cameras did not, while five papers reported that they did. A qualitative summary is available in Appendix 5. The three measures reported consistently to inform a quantitative analysis were subjective survey questions. These were: “Did participants notice the camera?”; “Did participants have concerns about the camera?”; and, “Did participants change their behaviour as a result of the camera?”.

**Fig. 2:**
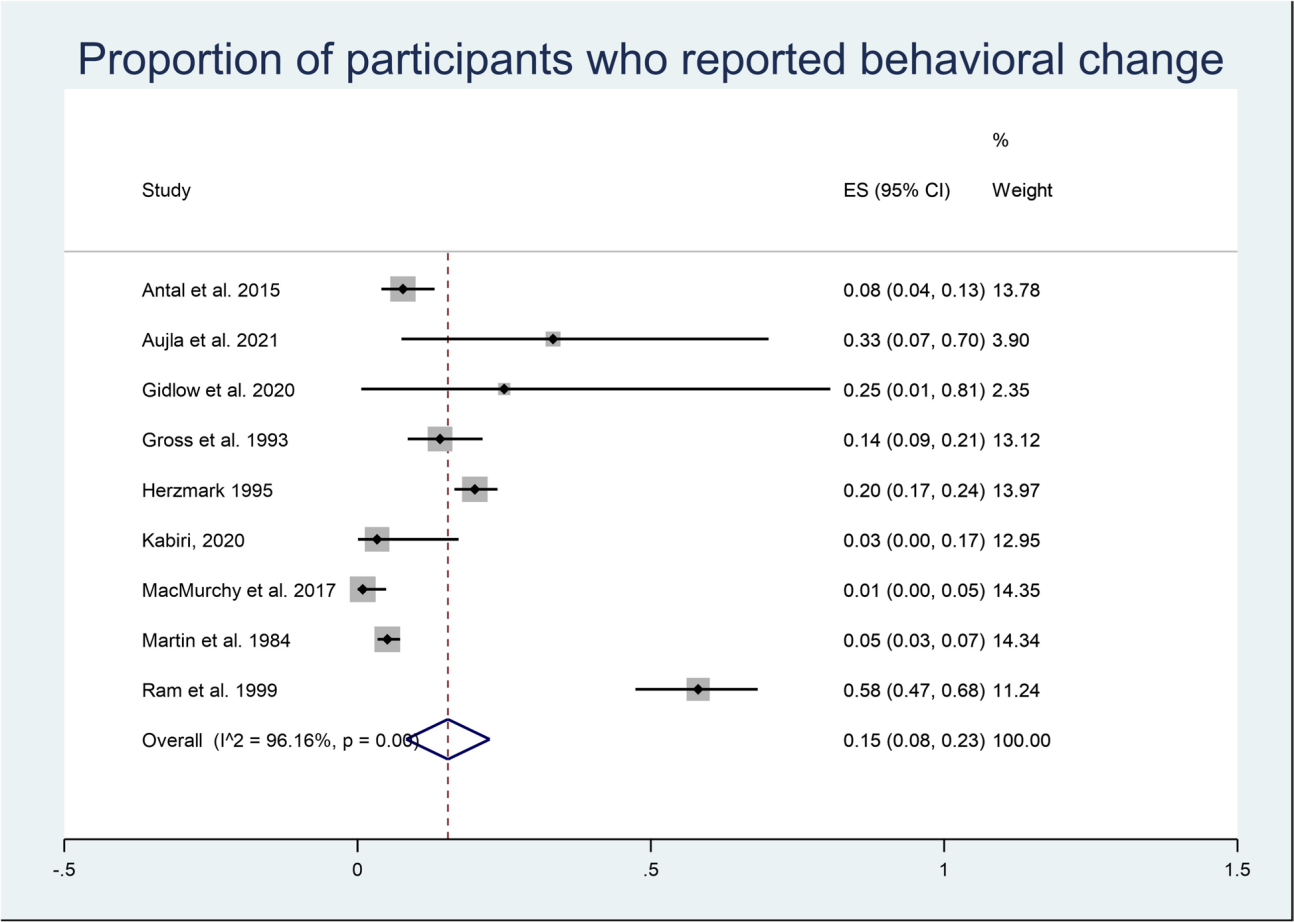
Meta-analysis participants reporting behavioural change

The proportion and 95% CI of participants who reported behavioural change were pooled across 9 studies using a random effects meta-analysis model (Fig. 2). Heterogeneity in the study estimates was assessed using the *I*^2^ statistic (96.16%) and Cochran’s *Q* *p* value(< 0.0001), which showed significant heterogeneity; thus, a random effects model was used, and the overall proportion of participants who reported behavioural change across the studies is 0.15 (95% CI 0.08, 0.23).

**Fig. 3:**
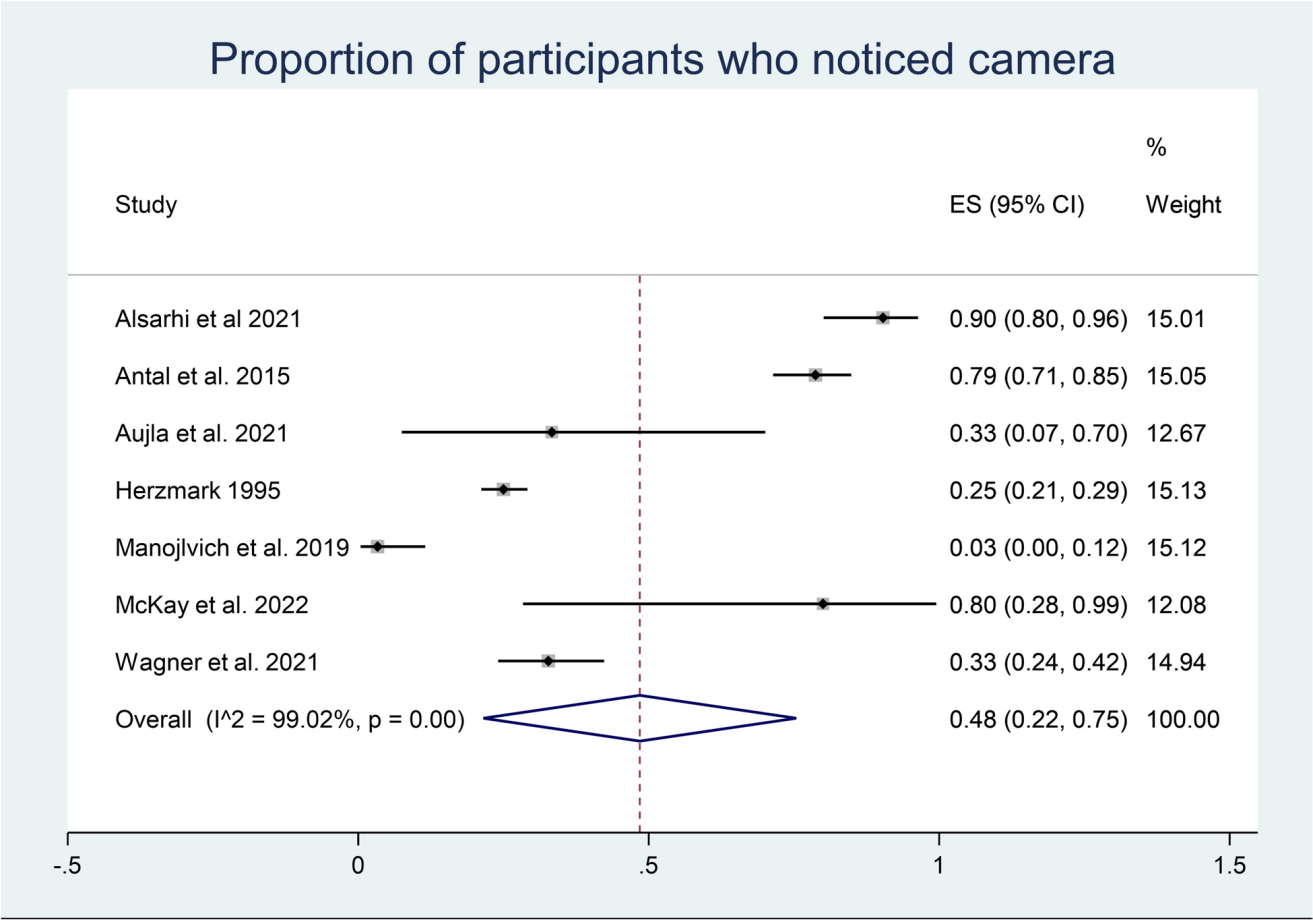
Meta-analysis participants noticing

**Fig. 4:**
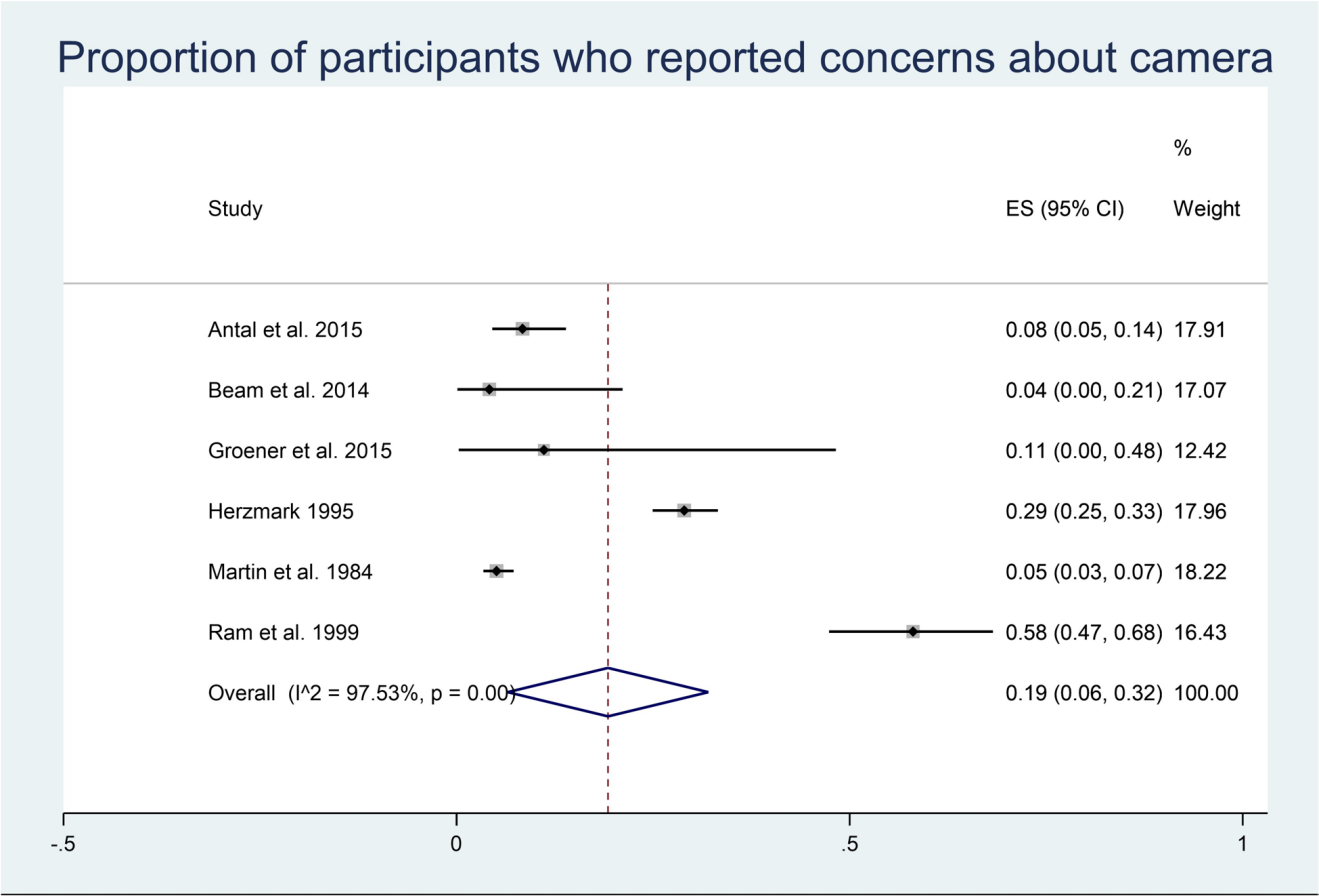
Meta-analysis participants concerned

The proportion and 95% CI of participants who noticed a camera when overtly filmed was pooled across seven studies using a random effects meta-analysis model (Fig. 3). Heterogeneity in the study estimates was assessed using the *I*^2^ statistic (99.02%) and Cochran’s *Q* *p* value(< 0.0001). A random effects model was used to not assume homogeneity of effects amongst studies. The overall proportion of participants who noticed the camera across the studies is 0.48 (95% CI 0.22, 0.75). The same process was used to determine the proportion of participants who reported concerns about the camera (Fig. 4). Heterogeneity in the study estimates was assessed using the *I*^2^ statistic (97.53%) and Cochran’s *Q p* value(< 0.0001). A random effects model was used, and the overall proportion of participants who reported a concern about the camera across the studies is 0.19 (95% CI 0.06, 0.32).

Six studies used objective methods to evaluate for presence or absence of The Hawthorne Effect [26, 28, 32, 42, 44, 48]. Tools used as measures were hand hygiene rates (*n* = 2) [32, 44], colonoscopy withdrawal time (*n* = 1) (48), and speed to complete a search task (*n* = 1) [42]. The two papers using hand hygiene rates reported conflicting results. Colonoscopy and search times suggested a change in behaviour due to camera reactivity. One publication used eye blink rate as a metric to determine the impact of awareness, [49] one publication coded all events that occurred on camera and evaluated what proportion of them were camera related, [26] and one examined rates of technical errors in donning personal protective equipment [28]. All three suggested that participants did not change their behaviour in response to the camera. 

Seven publications reported what sized camera was used [26, 28, 35, 38, 39, 42, 51]. They were classified as small (< 10 cm), medium (10–20 cm), or large (> 20 cm). Four papers reported using small cameras, of which all four suggested that the camera did not influence performance, comfort, or behaviour [28, 38, 39, 51]. The three papers that used medium-sized cameras had conflicting reports. Two suggested that the camera did not impact performance or comfort, but one found that it did [26, 35, 42]. 

Longer time spent in front of the camera increased comfort and reduced reactivity, as reported by six publications [27, 33, 34, 41, 43, 51]. All were subjective survey responses or individual participant comments such as “participants reported forgetting about the presence of the camera” [27].

Where data were missing, authors contacted corresponding authors via listed email for 24 publications; we received four responses, from which no further data was able to be included.

### Risk of bias

Two reviewers independently conducted a risk of bias assessment for each publication using the Newcastle–Ottawa Scale [53]. Any conflicts were resolved by discussion. Scores ranged between one and six of a possible nine stars across all publications. Full breakdown is available in Appendix 6.

## Discussion

The publications suggest that participants in studies that involve a video recording device are aware of the camera 48% of the time; they report feeling uncomfortable 19% of the time, and they change their behaviour 15% of the time, based on subjectively reported data. The results also suggest that time on camera could reduce participant discomfort and behavioural change related to filming. As this is the first quantitative analysis of this area, there are no directly comparable reviews. The heterogeneity in the reporting of the findings of each publication highlights the complexity of the topic in question.

Most humans have a desire to please or be seen to be kind [54]. There is evidence of this cognitive bias in everyday life [55]. This social desirability bias is often an unintended consequence that causes harm: a patient will not ‘trouble’ a clinician with an ailment; an athlete plays with an injury to support their team; or, a participant in research might change their behaviour to try and support the favoured outcome of the researcher. In research involving cameras, there can also be a spotlight effect [56]. The camera can activate objective self-awareness, where behaviour is compared against internal or social standards, and cause a participant to alter their behaviour away from a natural response [57]. A camera can imply judgement and permanence of record, which could trigger an anxiety-driven response [58]. These factors can further alter human behaviour in research studies; however, they have the potential to be mitigated by concealment of the recording device [3, 10]. 

An emerging concept that has the potential to reduce behavioural change we are witnessing is time and acclimation to the presence of the camera [27, 33, 34, 41, 43, 51]. Body camera wear in police was initially treated with scepticism as it was thought that people wearing the cameras would drastically alter their behaviour [59]. As their use becomes more prevalent, we see that acts of brutality and prejudice committed by or towards police are not deterred by these cameras [60]. Actors report that comfort with filming is primarily influenced by time spent in front of the lens. The more experience an actor has on camera, the less they are aware of it [61]. In the literature, there are suggestions that greater time spent on camera reduces participant behavioural change, but quantification or the implications of this phenomenon is difficult to determine [27, 33, 34, 41, 43, 51]. Not all studies lend themselves to allow an ‘acclimation’ time for participants, but the use of this may be a strategy that could reduce the impact of the Hawthorne Effect in future research.

The presence of a human observer during an experiment causes significant behavioural change [10, 14, 62]. This is highlighted in the recent observational research evaluating hand hygiene compliance amongst clinicians [9, 63–66]. There have been suggestions that the Hawthorne Effect can be exploited to improve performance in areas of medicine [15, 16, 67]. When objective criteria are used (e.g. hand hygiene compliance), 42.2% of participants change their behaviour as a result of awareness of a direct human observer [10, 12, 13, 68]. Relative to a direct observer, participant behavioural alteration due to a camera reduced by 27%. This figure should be interpreted with caution as it is not based on meta-analysis, and systematic reviews evaluating the Hawthorne Effect, to date, have not conducted a pooled estimate of this effect [10, 12, 13, 68]. The reasons for this are likely multifactorial. People are now subtly accustomed to the presence of a camera. Video doorbells, surveillance cameras, video conferencing, and camera phones are now a part of everyday life [22, 23]. Furthermore, modern cameras are tiny in comparison to the relics of the past. Technological age aside, a 20-year-old camera has a much smaller footprint than a human. A camera can be positioned to be out of a participant’s direct line of sight. A human, especially one who would not typically be present in a normal context, is much more difficult to conceal. Objects that occupy a smaller percentage of a visual field are less likely to be recognised, and therefore less likely to cause reactivity.

The heterogeneity of data included in this meta-analysis is likely due to variation in sample sizes, the small number of studies, and some extreme outliers [26, 27, 34, 36–38, 40, 46, 49]. This further highlights the challenges of assessing the Hawthorne Effect in research. Only six of the 28 publications utilised an objective outcome measure. Four publications used study-specific endpoints that cannot translate to other projects. Some were colonoscopy withdrawal time and hand hygiene compliance rate. One publication coded every single event, gesture, or action captured on camera then evaluated the proportion of ‘camera related behaviour’ in comparison to all other events. This relies heavily on the accuracy of coding and the context of other events that are being captured. The true validity and reliability of this process are unknown without further evaluation. In our current technological age, while computers and artificial intelligence are capable of extraordinary feats of processing, we are still incapable of this degree of sophisticated evaluation. It is possible that the use of deep learning can assist with this process, but first, an appropriate objective validated metric is required to enable the algorithm to accurately develop and grow.

Rates of camera awareness (48%) were much higher than rates of behavioural change (15%). Only two publications reported both [26, 37]. These studies reported lower rates of behavioural change in comparison with camera awareness. The publication using subjective results reported 80% of participants who noticed the camera changed their behaviour [37]. The other publication, which used objective measures to assess for behavioural change, reported 9.8% of the participants who noticed the camera also changed their behaviour [26]. This suggests that, in some cases, camera presence and awareness may be the cause of altered behaviour. The next consideration is how these actions impact the results of a study. The Hawthorne Effect could be directly related to the participant’s pre-participation study awareness. If a person sees a camera, it may cause a reaction, but it does not necessarily mean they react in a manner that compromises the results of a study. If a participant is unaware of the study goals, this limits their ability to ‘perform’ for the camera [69]. Tasks where participants are aware of the outcomes, such as hand hygiene rates, or conscious behavioural skill performances, are at high risk. If a participant is unaware of the outcome measure, such as a camera capturing rates of eye twitch in a double blinded pharmaceutical RCT, the results are less likely to be compromised by behavioural change [69]. Reactivity is greatest in research where participants are aware of the hypothesis [70]. Masking, or blinding, could reduce this phenomenon [70]. Similarly, if both cohorts are subject to the same conditions, the results are less likely to be impacted by the presence of a camera. If reactivity were to occur, both cohorts would still be comparable if conditions were identical [71].

## Strengths and limitations

This is the first meta-analysis evaluating the impact of video recording devices on participant behaviour in research. The comprehensive nature of the modified screening strategy minimised the risk of missing relevant publications where our study question was not the primary endpoint. This increased the overall sample size and generalisability of the findings. This study is an accurate representation of the evidence to date; however, interpretation of the findings must be done with consideration of the quality of evidence available. The majority of studies use subjective self-reported measures, as no universal tool to objectively evaluate the Hawthorne Effect with high validity and reliability exists. These results must be interpreted with caution due to the high risk of bias associated with each of the studies. This highlights the need for high-quality research into this field. The heterogeneity of the results made comparison between papers challenging, and publication bias could not be assessed due to limited data. Missing demographic data from publications further limits generalisability of the results and inhibited the ability to perform sensitivity analyses with regard to sex or cohort subtypes.

## Conclusion

The presence of a video camera may cause behavioural change in a small percentage of research participants. The degree to which this impacts results can possibly be mitigated by robust study design and blinding. Large, high-quality studies need to be conducted to determine if the phenomenon of The Hawthorne Effect exists in the face of evolving technology. As cameras give researchers a wealth of objective information with only 15% of participants changing their behaviour, their use should continue with care taken to design study methods that minimise their potential reactivity bias. Ethical use of camera concealment, subject habituation to observational conditions, and subject blinding to study endpoints may have the potential to mitigate the effects of The Hawthorne Effect.

## Supplementary Information

Supplementary Material 1: Appendix 1: MOOSE (Meta-analyses Of Observational Studies in Epidemiology) Checklist.

Supplementary Material 2: Appendix 2: MEDLINE search strategy.

Supplementary Material 3: Appendix 3: Studies excluded at full-text review.

Supplementary Material 4: Appendix 4: Data extraction plan.

Supplementary Material 5: Appendix 5: Qualitative summary.

Supplementary Material 6: Appendix 6: Risk of bias analysis.

Supplementary Material 7: Appendix 7: Studies included in meta-analysis.

## Data Availability

The datasets supporting the conclusions of this article are available in the Harvard dataverse repository, accessible via: https://dataverse.harvard.edu/privateurl.xhtml?token=a0192779-1b05-411b-8184-a3ad5a26a095. All authors accept responsibility for the decision to submit for publication. This study was a systematic review; thus, ethics approval was not required.
