# Supplementary material for: Evaluating the impact of video cameras on participant behaviour in research: a systematic review and meta-analysis
Source: Syst Rev. 2026 Jan 24;15:65. doi: 10.1186/s13643-025-03055-z (PMC12911182; doi:10.1186/s13643-025-03055-z)
Supplement: Supplementary file 2 — Supplementary Material 2: Appendix 2: MEDLINE search strategy. [file 13643_2025_3055_MOESM2_ESM.docx]

# MEDLINE Search Strategy

Database(s): **Ovid MEDLINE(R) ALL**1946 to October 31, 2022
Search Strategy:

| **#** | **Searches** | **Results** |
| --- | --- | --- |
| 1 | Awareness/ | 21646 |
| 2 | awareness.ti,ab,kf. | 189051 |
| 3 | ((aware* or reactiv* or act*1 or perform*) adj6 (camera* or watch* or video* or record* or observ* or monitor*)).ti,ab,kf. | 141383 |
| 4 | or/1-3 | 334625 |
| 5 | Behavior/ | 30167 |
| 6 | (behaviour or behavior).ti,ab,kf. | 1007519 |
| 7 | or/5-6 | 1020412 |
| 8 | and/4,7 | 22682 |
| 9 | Effect Modifier, Epidemiologic/ | 1400 |
| 10 | hawthorne effect*.ti,ab,kf. | 380 |
| 11 | observational reactivity.ti,ab,kf. | 1 |
| 12 | Camera Related Behavi*.ti,ab,kf. | 2 |
| 13 | or/8-12 | 24355 |
| 14 | exp Video Recording/ | 44242 |
| 15 | (video* audiovideo* or camera* or AV or recording).ti,ab,kf. | 198142 |
| 16 | or/14-15 | 237046 |
| 17 | Research Subjects/ | 6662 |
| 18 | ((research or trial or study) adj2 (participant* or subject*)).ti,ab,kf. | 131020 |
| 19 | Physician-Patient Relations/ | 75816 |
| 20 | ((physician* or clinician* or doctor*) adj2 (interaction* or consultation* or session*)).ti,ab,kf. | 5918 |
| 21 | ((physician* or clinician* or doctor*) adj3 patient* adj3 (communication* or interview or meeting or encounter*)).ti,ab,kf. | 8207 |
| 22 | doctor patient.ti,ab,kf. | 7550 |
| 23 | (clinical interaction* or clinical encounter or medical interaction).ti,ab,kf. | 2702 |
| 24 | exp Education, Professional/ | 325142 |
| 25 | exp Education, Premedical/ | 790 |
| 26 | exp Students, Health Occupations/ | 83930 |
| 27 | exp Simulation Training/ | 11303 |
| 28 | teach*.ti,ab,kf. | 224093 |
| 29 | educat*.ti,ab,kf. | 743757 |
| 30 | student*.ti,ab,kf. | 341876 |
| 31 | intern*1.ti,ab,kf. | 11829 |
| 32 | trainee*.ti,ab,kf. | 33451 |
| 33 | or/17-32 | 1456168 |
| 34 | and/13,16,33 | 160 |
