# Supplementary material for: Evaluating the impact of video cameras on participant behaviour in research: a systematic review and meta-analysis
Source: Syst Rev. 2026 Jan 24;15:65. doi: 10.1186/s13643-025-03055-z (PMC12911182; doi:10.1186/s13643-025-03055-z)
Supplement: Supplementary file 3 — Supplementary Material 3: Appendix 3: Studies excluded at full-text review. [file 13643_2025_3055_MOESM3_ESM.docx]

| Title | Authors | Year | Journal | Exclusion Reasons |
| --- | --- | --- | --- | --- |
| Motivation to learn: Can incentives improve laparoscopic training? | Abittan, B. S.; Grant, A.; Stauber, M.; Ricardo, A.; Nimaroff, M. | 2017 | Journal of minimally invasive gynecology | Wrong outcomes; |
| A behaviour assessment tool for simulated neonatal environment | Ahmed, A.; Moore, H.; Ranganna, R.; Didier, S.; Purva, M. | 2014 | Archives of Disease in Childhood | Wrong outcomes; |
| Influence of clinical communication on patients' decision making on participation in clinical trials | Albrecht, T. L.; Eggly, S. S.; Gleason, M. E.; Harper, F. W.; Foster, T. S.; Peterson, A. M.; Orom, H.; Penner, L. A.; Ruckdeschel, J. C.; Albrecht, Terrance L.; Eggly, Susan S.; Gleason, Marci E. J.; Harper, Felicity W. K.; Foster, Tanina S.; Peterson, Amy M.; Orom, Heather; Penner, Louis A.; Ruckdeschel, John C. | 2008 | Journal of clinical oncology | Wrong outcomes; |
| An observation tool for instructor and student behaviors to measure in-class learner engagement: a validation study | Alimoglu, M. K.; Sarac, D. B.; Alparslan, D.; Karakas, A. A.; Altintas, L. | 2014 | Medical Education Online | Wrong outcomes; |
| A comparison of three educational techniques used in a venereal disease clinic | Alkhateeb, W.; Lukeroth, C. J.; Riggs, M. | 1975 | Public health reports (Washington, D.C. : 1974) | Wrong outcomes; |
| Combining strategies to promote the generalization of self-management skills | Allen, R. | 2011 | Journal of Head Trauma Rehabilitation | Wrong study design; |
| The effects of organizational citizenship behavior on performance judgments: a field study and a laboratory experiment | Allen, T. D.; Rush, M. C. | 1998 | The Journal of applied psychology | Does not meet inclusion criteria ; |
| Development of a Child Abuse Checklist to Evaluate Prehospital Provider Performance | Alphonso, Aimee; Auerbach, Marc; Bechtel, Kirsten; Bilodeau, Kyle; Gawel, Marcie; Koziel, Jeannette; Whitfill, Travis; Tiyyagura, Gunjan Kamdar | 2017 | Prehospital Emergency Care | Wrong study design; |
| A Camera's view of consumer food-handling behaviors | Anderson, J. B.; Shuster, T. A.; Hansen, K. E.; Levy, A. S.; Volk, A. | 2004 | Journal of the American Dietetic Association | Does not meet inclusion criteria ; |
| The use of child actors and video clips to enhance learner immersion in paediatric emergency simulation | Ann, Lazarsfeld-Jensen; Amanda, Hlushak | 2013 | Australasian Journal of Paramedicine | Cant locate ; |
| Simulated patient and role play methodologies for communication skills and empathy training of undergraduate medical students | Bagacean, C.; Cousin, I.; Ubertini, A. H.; El Yacoubi El Idrissi, M.; Bordron, A.; Mercadie, L.; Garcia, L. C.; Ianotto, J. C.; De Vries, P.; Berthou, C. | 2020 | BMC medical education | Does not meet inclusion criteria ; |
| Non-technical skills for obstetricians conducting forceps and vacuum deliveries: qualitative analysis by interviews and video recordings | Bahl, R.; Murphy, D. J.; Strachan, B. | 2010 | European Journal of Obstetrics and Gynecology and Reproductive Biology | Does not meet inclusion criteria ; |
| Videotaping general practice consultations | Bain, J. E.; Mackay, N. S. | 1993 | BMJ | Wrong study design; |
| Computational analysis of the face and speech in acute psychosis and mania | Baker, J. | 2016 | Neuropsychopharmacology | Wrong study design; |
| Drama to promote social and personal well-being in six- and seven-year-olds with communication difficulties: the Speech Bubbles project | Barnes, Jonathan | 2014 | Perspectives in public health | Wrong study design; |
| Multiple realities in a study of medical consultations | Barry, C. A. | 2002 | Qualitative health research | Does not meet inclusion criteria ; |
| Investigating infection control behaviors in nurses | Beam, Elizabeth L. | 2014 |  | Wrong study design; |
| Movement characteristics of persons with Prader-Willi Syndrome rising from supine | Belt, A. B.; Hertel, T. A.; Mante, J. R.; Marks, T.; Rockett, V. L.; Wade, C.; Clayton-Krasinski, D. | 2001 | Pediatric Physical Therapy | Does not meet inclusion criteria ; |
| Escalation of resistiveness-to-care behaviors in people with dementia during hygienic care | Belzil, G.; Vezina, J. | 2013 | Alzheimer's and Dementia | Does not meet inclusion criteria ; |
| The impact of the Stanford Faculty Development Program on ambulatory teaching behavior | Berbano, E. P.; Browning, R.; Pangaro, L.; Jackson, J. L. | 2006 | Journal of general internal medicine | Does not meet inclusion criteria ; |
| Concordance Between Electronic Clinical Documentation and Physicians' Observed Behavior | Berdahl, Carl T.; Moran, Gregory J.; McBride, Owen; Santini, Alexandra M.; Verzhbinsky, Ilya A.; Schriger, David L. | 2019 | JAMA network open | Does not meet inclusion criteria ; |
| Patient disclosure of medical misdeeds | Bergen, C.; Stivers, T. | 2013 | Journal of Health and Social Behavior | Wrong outcomes; |
| A Prospective, Observational, Multicentre Study Concerning Nontechnical Skills in Robot-assisted Radical Cystectomy Versus Open Radical Cystectomy | Beulens, A. J. W.; Brinkman, W. M.; Koldewijn, E. L.; Hendrikx, A. J. M.; van Basten, J. P. A.; van Merrienboer, J. J. G.; Van der Poel, H. G.; Bangma, C. H.; Wagner, C. | 2020 | European Urology Open Science | Wrong outcomes; |
| The assessment of water-use behaviours after implementation of new water infrastructure at a remote Himalayan school | Bhatla, C.; Soni, D.; Bhatla, J.; Raff, D.; Skutezky, T.; Fairley, J.; Chung, J.; Herman, J.; Kapoor, V. | 2016 | Annals of global health | Wrong outcomes; |
| Comprehensive surgical coaching enhances surgical skill in the operating room: A randomized controlled trial | Bonrath, E. M.; Dedy, N. J.; Gordon, L. E.; Grantcharov, T. P. | 2015 | Annals of surgery | Wrong outcomes; |
| Development and piloting the Woman Centred Care Scale (WCCS) | Brady, Susannah; Bogossian, Fiona; Gibbons, Kristen | 2017 | Women & Birth | Wrong outcomes; |
| Memory of psychodiagnostic information: biases and effects of expertise | Brailey, K.; Vasterling, J. J.; Franks, J. J. | 2001 | The American journal of psychology | Wrong study design; |
| Resident reactions to unannounced standardized patients in the ED | Brazg, J.; Chung, A.; Retino, C.; Marshall, J.; Saloum, D. | 2016 | Western Journal of Emergency Medicine | Wrong outcomes; |
| A simulator-based tool that assesses pediatric resident resuscitation competency | Brett-Fleegler, M. B.; Vinci, R. J.; Weiner, D. L.; Harris, S. K.; Shih, M.; Kleinman, M. E. | 2008 | Pediatrics | Wrong outcomes; |
| Nursing Faculty Beliefs and Practices Regarding Debriefing Human Patient Simulation Experiences | Brown, Francesca Spano | 2011 |  | Wrong outcomes; |
| Improving self-management in cleaning equipment in 10-13 year olds with cf | Browning, G.; Cooley, R.; Stamper, S.; Filigno, S. S.; Miller, J. L.; Moore, S.; Mullen, L.; Siracusa, C.; Weiland, J.; Burns, L. A. | 2016 | Pediatric pulmonology | Wrong outcomes; |
| Clinical performance assessment and interactive video teleconferencing: an iterative exploration | Bulik, R. J.; Frye, A. W.; Callaway, M. R.; Romero, C. M.; Walters, D. J. | 2002 | Teaching and learning in medicine | Wrong outcomes; |
| Exploration of Two Methodologies for Measuring Clinical Judgment in Baccalaureate Nursing Students | Call, Marlene W. | 2017 | Exploration of Two Methodologies for Measuring Clinical Judgment in Baccalaureate Nursing Students | Wrong study design; |
| Evaluation of neonatal resuscitation using high fidelity simulation and an interprofesional team | Campbell, D.; Ying, E.; Kin Fan, Y. T.; Sgro, M.; Barozzino, T. | 2010 | Paediatrics and Child Health | Wrong outcomes; |
| In Vivo Feedback Predicts Parent Behavior Change in the Attachment and Biobehavioral Catch-up Intervention | Caron, E. B.; Bernard, K.; Dozier, M. | 2018 | Journal of clinical child and adolescent psychology : the official journal for the Society of Clinical Child and Adolescent Psychology, American Psychological Association, Division 53 | Wrong outcomes; |
| Assessment of non-technical skills in acute care team simulation training | Cha, J. S.; Anton, N. E.; Mizota, T.; Hennings, J. M.; Rendina, M. A.; Stanton-Maxey, K.; Ritter, H. E.; Stefanidis, D.; Yu, D. | 2018 | Surgical Endoscopy and Other Interventional Techniques | Wrong outcomes; |
| Action-related fixation in microsuturing, a new gaze behavior metric to differentiate the level of expertise | Chainey, J.; Zheng, B.; Kim, M.; Elomaa, A.; Bednarik, R.; O'Kelly, C. | 2022 | Canadian Journal of Neurological Sciences | Wrong outcomes; |
| Cue-responding during simulated routine nursing care: A mixed method study | Chan, Engle A. | 2014 | Nurse Education Today | Wrong outcomes; |
| New simulation-based airway management training program for junior physicians: Advanced Airway Life Support | Chen, P.; Huang, Y.; Cheng, H.; Wang, C.; Chan, C.; Chan, K.; Kuo, C. | 2009 | Medical teacher | Wrong outcomes; |
| Training residents to C.A.R.E. using videotaped unannounced standardized patient encounters | Chung, A.; Saloum, D.; Retino, C.; Brazg, J.; Weiner, C.; Pushkar, I.; Drapkin, J.; Likourezos, A.; Marshall, J. | 2017 | Western Journal of Emergency Medicine | Wrong outcomes; |
| How do examiners' personal epistemologies affect their assessment decisions in an OSCE setting? | Coulby, C.; Fuller, R.; Jha, V. | 2010 | Medical Education, Supplement | Wrong study design; |
| An instrument for assessment of videotapes of general practitioners' performance | Cox, J.; Mulholland, H. | 1993 | British Medical Journal | Wrong study design; |
| Teaching strategies: a microteaching project for nurses in Virginia | Crosby, M. H. | 1977 | Nursing Research | Wrong outcomes; |
| Video-based Self-reflection - A New Tool in an Innovative Teaching Concept, Adapted to the Topic "Interprofessional Collaboration in Acute In-patient Rehabilitatione" | Dahmen, U.; Loudovici-Krug, D.; Schulze, C.; Veit, A.; Eiselt, M.; Smolenski, U. C. | 2015 | Physikalische Medizin Rehabilitationsmedizin Kurortmedizin | Wrong outcomes; |
| General practitioners apply the usual care for shoulder complaints better than expected -- analysis of videotaped consultations | De Bruijn, C.; de Bie, R.; Geraets, J.; Goossens, M.; KÃ¶ke, A.; van den Heuvel, W.; Dinant, G. | 2007 | BMC family practice | Wrong outcomes; |
| Anxiety and performance of nursing students in regard to assessment via clinical simulations in the classroom versus filmed assessments | de Souza Teixeira, C. R.; Kusumota, L.; Alves Pereira, M. C.; Merizio Martins Braga, F. T.; Pirani Gaioso, V.; Mara Zamarioli, C.; Campos de Carvalho, E. | 2014 | Investigacion y educacion en enfermeria | Wrong outcomes; |
| Toothbrushing behavior in children - an observational study of toothbrushing performance in 12 year olds | Deinzer, R.; Cordes, O.; Weber, J.; Hassebrauck, L.; Weik, U.; Kramer, N.; Pieper, K.; Margraf-Stiksrud, J. | 2019 | BMC Oral Health | Wrong outcomes; |
| Education, monitoring and outcome in providers of a self-regulation program to increase physical activity in rheumatoid arthritis | Demmelmaier, I. | 2013 | Annals of the Rheumatic Disease | Wrong outcomes; |
| Feasibility of DAIly NURSE: A nursing intervention to change nursing staff behaviour towards encouraging residents' daily activities and independence in the nursing home | den Ouden, M.; Zwakhalen, S. M. G.; Meijers, J. M. M.; Bleijlevens, M. H. C.; Hamers, J. P. H. | 2019 | Journal of clinical nursing | Wrong outcomes; |
| An observational study using eye tracking to assess resident and senior anesthetists' situation awareness and visual perception in postpartum hemorrhage high fidelity simulation | Desvergez, A.; Winer, A.; Gouyon, J. B.; Descoins, M. | 2019 | PloS one | Wrong outcomes; |
| Evaluation of a Measurement System to Assess ICU Team Performance | Dietz, Aaron S.; Salas, Eduardo; Pronovost, Peter J.; Jentsch, Florian; Wyskiel, Rhonda; Mendez-Tellez, Pedro Alejandro; Dwyer, Cynthia; Rosen, Michael A. | 2018 | Critical care medicine | Wrong outcomes; |
| Videorecording of experts as a method of training-simulator design | Doozandeh, Pooyan | 2022 | Theoretical Issues in Ergonomics Science | Wrong study design; |
| 'GP Live'- recorded General Practice consultations as a learning tool for junior medical students faced with the COVID-19 pandemic restrictions | Dow, N.; Wass, V.; Macleod, D.; Muirhead, L.; McKeown, J. | 2020 | Education for primary care : an official publication of the Association of Course Organisers, National Association of GP Tutors, World Organisation of Family Doctors | Wrong outcomes; |
| Gross motor function and activity in rett syndrome | Downs, J. | 2016 | Wiener Medizinische Wochenschrift | Wrong outcomes; |
| Automatic Surgical Skill Rating Using Stylistic Behavior Components | Ershad, M.; Rege, R.; Fey, A. M. | 2018 | Conference proceedings : ... Annual International Conference of the IEEE Engineering in Medicine and Biology Society. IEEE Engineering in Medicine and Biology Society. Annual Conference | Wrong outcomes; |
| Evaluation of a digitized physician-patient-communication course evaluated by preclinical medical students: a replacement for classroom education? | Fischbeck, S.; Hardt, J.; Malkewitz, C.; Petrowski, K. | 2020 | GMS journal for medical education | Wrong outcomes; |
| Optimal format to assess intervention fidelity in mindfulness research | Floyd, E.; Adler, S.; Moran, P.; Hartogensis, W.; Richler, R.; Crane, R.; Brewer, J.; Hecht, F. | 2020 | Global advances in health and medicine | Wrong Outcomes |
| Surgical skills: Can learning curves be computed from recordings of surgical activities? | Forestier, G.; Riffaud, L.; Petitjean, F.; Henaux, P. L.; Jannin, P. | 2018 | International Journal of Computer Assisted Radiology and Surgery | Wrong outcomes; |
| Teaching communication skills in an integrated curriculum | Friedrich, R. M.; Lively, S. I.; Schacht, E. | 1985 | The Journal of nursing education | Wrong study design; |
| Developing of distributed case discussion support system for analyzing the behavioural factors of ASD people | Fujii, Y.; Ogawa, H.; Takano, M. | 2012 | Journal of Intellectual Disability Research | Wrong outcomes; |
| Assessment of clinical performance during simulated crises using both technical and behavioral ratings | Gaba, D. M.; Howard, S. K.; Flanagan, B.; Smith, B. E.; Fish, K. J.; Botney, R. | 1998 | Anesthesiology | Wrong outcomes; |
| Using Multitouch Collaboration Technology to Enhance Social Interaction of Children with High-Functioning Autism | Gal, E.; Lamash, L.; Bauminger-Zviely, N.; Zancanaro, M.; Weiss, P. L. | 2016 | Physical & occupational therapy in pediatrics | Wrong outcomes; |
| The effects of intranasal oxytocin on contagious yawning | Gallup, A. C.; Church, A. M. | 2015 | Neuroscience Letters | Wrong outcomes; |
| Physiological and self-assessed psychological stress induced by a high fidelity simulation course among third year anesthesia and critical care residents: An observational study | Geeraerts, T.; Roulleau, P.; Cheisson, G.; Marhar, F.; Aidan, K.; Lallali, K.; Leguen, M.; Schnell, D.; Trabold, F.; Fauquet-Alekhine, P.; Duranteau, J.; Benhamou, D. | 2017 | Anaesthesia Critical Care and Pain Medicine | Wrong outcomes; |
| Microteaching and standardized students support faculty development for clinical teaching | Gelula, M. H.; Yudkowsky, R. | 2002 | Academic medicine : journal of the Association of American Medical Colleges | Wrong outcomes; |
| Using standardised students in faculty development workshops to improve clinical teaching skills | Gelula, M. H.; Yudkowsky, R. | 2003 | Medical education | Wrong outcomes; |
| Measuring physician behavior | Gerbert, B.; Hargreaves, W. A. | 1986 | Medical care | Wrong outcomes; |
| Which symptoms matter? Self-report and observer discrepancies in repressors and high-anxious women with metastatic breast cancer | Giese-Davis, Janine; Tamagawa, Rie; Yutsis, Maya; Twirbutt, Suzanne; Piemme, Karen; Neri, Eric; Taylor, C.; Spiegel, David | 2014 | Journal of Behavioral Medicine | Wrong outcomes; |
| Primary care doctors' needs for information: Analysis of questions and how they were answered | Gonzalez-Gonzalez, A. I.; Escortell Mayor, E.; Hernandez Fernandez, T.; Sanchez Mateos, J. F.; Sanz Cuesta, T.; Riesgo Fuertes, R.; Domenech Senra, P.; Fernandez San Martin Ma, I.; Cabello Ballesteros, L.; Silva Mato, A.; Munoz Garcia, J. C.; Moros, J. Ma M. | 2005 | Atencion Primaria | Wrong outcomes; |
| Does simulator-based clinical performance correlate with actual hospital behavior? The effect of extended work hours on patient care provided by medical interns | Gordon, J. A.; Alexander, E. K.; Lockley, S. W.; Flynn-Evans, E.; Venkatan, S. K.; Landrigan, C. P.; Czeisler, C. A.; Harvard Work Hours, Health; Safety, Group | 2010 | Academic medicine : journal of the Association of American Medical Colleges | Wrong outcomes; |
| Facilitating error recognition and patient safety awareness in pre-registration physiotherapy students using video reflexive ethnography and simulation-based education | Gough, S.; Yohannes, A.; Roberts, P.; Murrray, J.; Sixsmith, J. | 2015 | Physiotherapy (united kingdom) | Wrong outcomes; |
| Longitudinal resident coaching in the ambulatory setting: A novel intervention to improve clinical skills | Graddy, R.; Reynolds, S.; Wright, S. | 2019 | Journal of general internal medicine | Wrong outcomes; |
| Live streaming to sustain clinical learning | Grafton-Clarke, C.; Uraiby, H.; Abraham, S.; Kirtley, J.; Xu, G.; McCarthy, M. | 2022 | The clinical teacher | Wrong study design; |
| Do medical students and young physicians assess reliably their self-efficacy regarding communication skills? A prospective study from end of medical school until end of internship | Gude, T.; Finset, A.; Anvik, T.; Baerheim, A.; Fasmer, O. B.; Grimstad, H.; Vaglum, P. | 2017 | BMC medical education | Wrong outcomes; |
| Long-Term Outcomes of a Simulation-Based Remediation for Residents and Faculty With Unprofessional Behavior | Guerrasio, J.; Aagaard, E. M. | 2018 | Journal of graduate medical education | Wrong outcomes; |
| Methods to improve reliability of video-recorded behavioral data | Haidet, K. K.; Tate, J.; Divirgilio-Thomas, D.; Kolanowski, A.; Happ, M. B. | 2009 | Research in nursing & health | Wrong study design; |
| Observer-rated rapport in interactions between medical students and standardized patients | Hall, J. A.; Roter, D. L.; Blanch, D. C.; Frankel, R. M. | 2009 | Patient Education & Counseling | Wrong outcomes; |
| Nursing home care: changes after supervision | Hansebo, G.; Kihlgren, M. | 2004 | Journal of Advanced Nursing (Wiley-Blackwell) | Wrong outcomes; |
| What is the best predictor for oral cleanliness after brushing? Results from an observational cohort study | Harnacke, Daniela; Winterfeld, Tobias; Erhardt, JÃ¶rg; Schlueter, Nadine; Ganss, Carolina; Margraf-Stiksrud, Jutta; Deinzer, Renate | 2015 | Journal of Periodontology | Wrong outcomes; |
| Social anxiety and self-impression: Cognitive preparation enhances the beneficial effects of video feedback following a stressful social task | Harvey, A. G.; Clark, D. M.; Ehlers, A.; Rapee, R. M. | 2000 | Behaviour Research and Therapy | Wrong study design; |
| Collaborative performance in laparoscopic teams: behavioral evidences from simulation | He, W.; Zheng, B. | 2016 | Surgical Endoscopy | Wrong outcomes; |
| Effect of clinician feedback versus video self-assessment in 5th-year chiropractic students on an end-of-year communication skills examination | Hecimovich, M. D.; Maire, J.; Losco, B. | 2010 | Journal of Chiropractic Education (Association of Chiropractic Colleges) | Does not meet inclusion criteria ; |
| Video analyses discovery awareness | Heijkoop, J.; Clegg, J.; Webb, J. | 2015 | Journal of Intellectual Disability Research | Wrong study design; |
| Use of Immersive Learning and Simulation Techniques to Teach and Research Opioid Prescribing Practices | Heirich, Marissa S.; Sinjary, Lanja S.; Ziadni, Maisa S.; Sacks, Sandra; Buchanan, Alexandra S.; Mackey, Sean C.; Newmark, Jordan L. | 2019 | Pain Medicine | Wrong outcomes; |
| The role of ethnicity and socioeconomic status in Southeast Asian mothers' parenting sensitivity | Heng, J.; Quan, J.; Sim, L. W.; Sanmugam, S.; Broekman, B.; Bureau, J. F.; Meaney, M. J.; Holbrook, J. D.; Rifkin-Graboi, A. | 2018 | Attachment & Human Development | Wrong outcomes; |
| Gender-focused training improves leadership of female medical students: A randomized trial | Hochstrasser, S. R.; Amacher, S. A.; Tschan, F.; Semmer, N. K.; Becker, C.; Metzger, K.; Hunziker, S.; Marsch, S. | 2021 | Medical education | Wrong outcomes; |
| Teaching concepts of clinical measurement variation to medical students | Hodder, R. A.; Longfield, J. N.; Cruess, D.; Horton, J. A. | 1982 | International Journal of Epidemiology | Does not meet inclusion criteria ; |
| Prevention of back pain in hospitals, new approaches to movement-consciousness | Hohnke, O.; Stratmann, A.; Haamann, F.; Schramm, J. | 1997 | Arbeitsmedizin Sozialmedizin Umweltmedizin | Incomplete dataset; |
| Effects of training in direct observation of medical resident's clinical competence: A randomized trial | Holmboe, E. S.; Hawkins, R. E.; Huot, S. J. | 2004 | Annals of internal medicine | Wrong study design; |
| Simulation study of rested versus sleep-deprived anesthesiologists | Howard, S. K.; Gaba, D. M.; Smith, B. E.; Weinger, M. B.; Herndon, C.; Keshavacharya, S.; Rosekind, M. R.; Howard, Steven K.; Gaba, David M.; Smith, Brian E.; Weinger, Matthew B.; Herndon, Christopher; Keshavacharya, Shanthala; Rosekind, Mark R. | 2003 | Anesthesiology | Wrong outcomes; |
| Detecting psychological distress: can general practitioners improve their own performance? | Howe, A. | 1996 | British journal of general practice | Wrong study design; |
| Is seeing believing? technical mentorship during robot-assisted surgery | Hussein, A.; Shafiei, S.; Sharif, M.; Ahmad, B.; Esfahani, E.; Guru, K. | 2016 | Journal of urology | Wrong outcomes; |
| The effects of cognitive restructuring on assertive behavior | Jacobs, M. K.; Cochran, S. D. | 1982 | Cognitive therapy and research | Wrong outcomes; |
| Measuring team performance in healthcare: review of research and implications for patient safety | Jeffcott, S. A.; Mackenzie, C. F. | 2008 | Journal of Critical Care | Wrong study design; |
| Self-assessment via videotaping to maximize teaching effectiveness | Jeffers, J. M.; Guthrie, D. W. | 1988 | Journal of continuing education in nursing | Wrong outcomes; |
| Addressing the chronically unexplained: Using standardized patients to teach medical residents pragmatic adverse childhood experience (ACE) based interventions | Jelley, M.; Miller-Cribbs, J.; Oberst-Walsh, L.; Rodriguez, K.; Wen, F. K.; Coon, K. | 2016 | Journal of general internal medicine | Wrong outcomes; |
| Children with PIMD: Intervention study targeting school staffs' responsive strategies using interactive music activities and multisensory storytelling | Johnels, L.; Wilder, J.; Vehmas, S. | 2019 | Journal of Intellectual Disability Research | Wrong outcomes; |
| High-performing trauma teams: frequency of behavioral markers of a shared mental model displayed by team leaders and quality of medical performance | Johnsen, B. H.; Westli, H. K.; Espevik, R.; Wisborg, T.; Brattebo, G. | 2017 | Scandinavian journal of trauma, resuscitation and emergency medicine | Wrong outcomes; |
| Internal and External Focus of Attention During Gait Re-Education: An Observational Study of Physical Therapist Practice in Stroke Rehabilitation | Johnson, Louise; Burridge, Jane H.; Demain, Sara H. | 2013 | Physical therapy | Wrong outcomes; |
| Self-reported changes in clinical behaviour by undergraduate dental students after video-based teaching in paediatric dentistry | Kalwitzki, M. | 2005 | European journal of dental education : official journal of the Association for Dental Education in Europe | Wrong outcomes; |
| Assessing clinicians' consultation with people with profound learning disability: producing a rating scale | Kerr, M. P.; Evans, S.; Nolan, M.; Fraser, W. I. | 1995 | Journal of Intellectual Disability Research | Wrong outcomes; |
| Remote Video Auditing (RVA) To Assess Personal Protective Equipment (PPE) Compliance In Rooms With Clostridioides difficile Patients | Khatri, A. M.; Khameraj, A.; Franklin, T.; Malhotra, P.; Farber, B. | 2020 | Open forum infectious diseases | Does not meet inclusion criteria ; |
| Reactivity in self-recording: obtrusiveness of recording procedure and peer comments | Kirby, K. C.; Fowler, S. A.; Baer, D. M. | 1991 | Journal of Applied Behavior Analysis | Wrong setting; |
| Reâ€viewing performance: Showing eyeâ€tracking data as feedback to improve performance monitoring in a complex visual task | Kok, Ellen; Hormann, Olle; Rou, Jeroen; van Saase, Evi; van der Schaaf, Marieke; Kester, Liesbeth; van Gog, Tamara | 2022 | Journal of Computer Assisted Learning | Wrong study design; |
| Speaking up is related to better team performance in simulated anesthesia inductions: An observational study | Kolbe, M.; Burtscher, M. J.; Wacker, J.; Grande, B.; Nohynkova, R.; Manser, T.; Spahn, D. R.; Grote, G. | 2012 | Anesthesia and analgesia | Wrong outcomes; |
| Utilizing Grasp Monitoring to Predict Microsurgical Expertise | Koskinen, J.; He, W.; Elomaa, A. P.; Kaipainen, A.; Hussein, A.; Zheng, B.; Huotarinen, A.; Bednarik, R. | 2023 | Journal of Surgical Research | Wrong outcomes; |
| Joint Attention in a Laparoscopic Simulation-Based Training: A Pilot Study on Camera Work, Gaze Behavior, and Surgical Performance in Laparoscopic Surgery | Krois, W.; Reck-Burneo, C. A.; Gropel, P.; Wagner, M.; Berger, A.; Metzelder, M. L. | 2020 | Journal of Laparoendoscopic and Advanced Surgical Techniques | Wrong outcomes; |
| Face touching: A frequent habit that has implications for hand hygiene | Kwok, Yen Lee Angela; Gralton, Jan; McLaws, Mary-Louise | 2015 | American Journal of Infection Control | Wrong outcomes; |
| Brief report: Teaching persons with moderate mental retardation 10 use printed instructions and time cues in a work context | Lancioni, G. E.; Van den Hof, E.; Brouwer, J. A. | 1995 | Behavioral Interventions | Wrong outcomes; |
| An evidence based framework for the Temporal Observational Analysis of Teamwork in healthcare settings | Lavelle, Mary; Reedy, Gabriel B.; Cross, Sean; Jaye, Peter; Simpson, Thomas; Anderson, Janet E. | 2020 | Applied Ergonomics | Wrong outcomes; |
| Observing executive activity in a simple assembly task | Lawson, M. J. | 1985 | American journal of mental deficiency | Wrong outcomes; |
| The impact of expert visual guidance on trainee visual search strategy, visual attention and motor skills | Leff, D. R.; James, D. R. C.; Orihuela-Espina, F.; Kwok, K. W.; Sun, L. W.; Mylonas, G.; Athanasiou, T.; Darzi, A. W.; Yang, G. Z. | 2015 | Frontiers in human neuroscience | Wrong outcomes; |
| Doctor-couple communication during assisted reproductive technology visits | Leone, D.; Borghi, L.; Del Negro, S.; Becattini, C.; Chelo, E.; Costa, M.; De Lauretis, L.; Ferraretti, A. P.; Giuffrida, G.; Livi, C.; Luehwink, A.; Palermo, R.; Revelli, A.; Tomasi, G.; Tomei, F.; Filippini, C.; Vegni, E. | 2018 | Human Reproduction | Wrong outcomes; |
| A Pilot Comparison of In-Room and Video Ratings of Team Behaviors of Students in Interprofesional Teams | Lie, DÃ©sirÃ©e; Richter-Lagha, Regina; Ma, Sae Byul | 2018 | American journal of pharmaceutical education | Wrong outcomes; |
| Comparison of surgical skill acquisition by UK surgical trainees and Sierra Leonean associate clinicians in a task-sharing programme | Liu, B.; Hunt, L. M.; Lonsdale, R. J.; Narula, H. S.; Mansaray, A. F.; Bundu, I.; Bolkan, H. A. | 2019 | BJS Open | Wrong outcomes; |
| Development of a novel empathy-related video-feedback intervention to improve empathic accuracy of nursing students: A pilot study | Lobchuk, Michelle; Halas, Gayle; West, Christina; Harder, Nicole; Tursunova, Zulfiya; Ramraj, Chantal | 2016 | Nurse Education Today | Wrong outcomes; |
| Assessment of the Hawthorne effect during central venous catheter manipulation | Lobo, R. D.; Oliveira, M. S.; Colella, J. J.; Silva, N. D. D.; Pastore Junior, L.; Souza, R. C. D. S. | 2022 | Revista da Escola de Enfermagem da U S P | Wrong study design; |
| Following the rules: Consistency in sign | Luetke-Stahlman, B. | 1991 | Journal of Speech and Hearing Research | Wrong outcomes; |
| Observation patterns of dynamic occupational performance | MacKenzie, D. E.; Westwood, D. A. | 2013 | Canadian journal of occupational therapy. Revue canadienne d'ergotherapie | Wrong study design; |
| Differences in Faculty and Standardized Patient Scores on Professionalism for Second-Year Podiatric Medical Students During a Standardized Simulated Patient Encounter | Mahoney, James M.; Vardaxis, Vassilios; Anwar, Noreen; Hagenbucher, Jacob | 2018 | Journal of the American Podiatric Medical Association | Wrong outcomes; |
| Physician gender affects how physician nonverbal behavior is related to patient satisfaction | Mast, M. S.; Hall, J. A.; KlÃ¶ckner, C.; Choi, E. | 2008 | Medical care | Wrong outcomes; |
| Be SMARTT about trauma: An interdisciplinary educational approach to improving teamwork in the trauma bay | McDowell, C. M.; Roberts, N. K.; Sutyak, J.; Griffen, D.; Wall, J.; Schwind, C.; Williams, R. G. | 2012 | Annals of emergency medicine | Wrong outcomes; |
| Leaders' and followers' individual experiences during the early phase of simulation-based team training: an exploratory study | Meurling, Lisbet; Hedman, Leif; FellÃ¤nder-Tsai, Li; Wallin, Carl-Johan | 2013 | BMJ quality & safety | Wrong outcomes; |
| Assessing neurosurgical non-technical skills: An exploratory study of a new behavioural marker system | Michinov, E.; Jamet, E.; Dodeler, V.; Haegelen, C.; Jannin, P. | 2014 | Journal of evaluation in clinical practice | Wrong outcomes; |
| Identifying key nursing and team behaviours to achieve high reliability | Miller, Kristi; Riley, William; Davis, Stanley | 2009 | Journal of Nursing Management (Wiley-Blackwell) | Wrong outcomes; |
| Speaking across the drapes: communication strategies of anesthesiologists and obstetricians during a simulated maternal crisis | Minehart, R. D.; Pian-Smith, M. C.; Walzer, T. B.; Gardner, R.; Rudolph, J. W.; Simon, R.; Raemer, D. B. | 2012 | Simulation in healthcare : journal of the Society for Simulation in Healthcare | Wrong outcomes; |
| Learning effect of a novel interactive basic life support CD: The JUST system | Monsieurs, K. G.; Vogels, C.; Bossaert, L. L.; Meert, P.; Manganas, A.; Tsiknakis, M.; Leisch, E.; Calle, P. A.; Giorgini, F. | 2004 | Resuscitation | Wrong study design; |
| Task management skills and their deficiencies during care delivery in simulated medical emergency situation: A classification | Morineau, Thierry; Chapelain, Pascal; Quinio, Philippe | 2016 | Intensive & Critical Care Nursing | Wrong outcomes; |
| A REVIEW OF VIDEO REVIEW: NEW PROCESSES FOR THE 21ST CENTURY | Muench, John; Sanchez, Demetrio; Garvin, Roger | 2013 | International journal of psychiatry in medicine | Wrong study design; |
| Evaluation of a shared decision-making communication skills training for physicians treating patients with asthma: a mixed methods study using simulated patients | Muller, E.; Diesing, A.; Rosahl, A.; Scholl, I.; Harter, M.; Buchholz, A. | 2019 | BMC health services research | Wrong outcomes; |
| Evaluation of multidisciplinary simulation training on clinical performance and team behavior during tracheal intubation procedures in a pediatric intensive care unit | Nishisaki, Akira; Nguyen, Joan; Colborn, Shawn; Watson, Christine; Niles, Dana; Hales, Roberta; Devale, Sujatha; Bishnoi, Ram; Nadkarni, Lindsay D.; Donoghue, Aaron J.; Meyer, Andrew; Brown 3rd, Calvin a; Helfaer, Mark A.; Boulet, John; Berg, Robert A.; Walls, Ron M.; Nadkarni, Vinay M. | 2011 | Pediatric Critical Care Medicine | Does not meet inclusion criteria ; |
| Discussing patient's lifestyle choices in the consulting room: analysis of GP-patient consultations between 1975 and 2008 | Noordman, Janneke; Verhaak, Peter; van Dulmen, Sandra | 2010 | BMC family practice | Wrong outcomes; |
| Evaluation of developmental surveillance by physicians at the two-month preventive care visit | Nyp, S. S.; Barone, V. J.; Kruger, T.; Garrison, C. B.; Robertsen, C.; Christophersen, E. R. | 2011 | Journal of Applied Behavior Analysis | Wrong outcomes; |
| A resident's training program for the development of smoking intervention skills | Ockene, J. K.; Quirk, M. E.; Goldberg, R. J.; Kristeller, J. L.; Donnelly, G.; Kalan, K. L.; Gould, B.; Greene, H. L.; Harrison-Atlas, R.; Pease, J.; Pickens, S.; Williams, J. W. | 1988 | Archives of internal medicine | Wrong outcomes; |
| The use of digital technology to improve and monitor handwashing among children 12 years or younger in educational settings: a systematic review | Ofori, S. K.; Hung, Y. W.; Muniz-Rodriguez, K.; Kakau, R. J.; Alade, S. E.; Diallo, K.; Sullivan, K. L.; Schwind, J. S.; Cowling, B. J.; Fung, I. C. H. | 2019 | American Journal of Tropical Medicine and Hygiene | Wrong study design; |
| Temporal characteristics of decisions in hospital encounters: A threshold for shared decision making? A qualitative study | Ofstad, E. H.; Frich, J. C.; Schei, E.; Frankel, R. M.; Gulbrandsen, P. | 2014 | Patient education and counseling | Wrong outcomes; |
| Effectiveness of remote simulation-based learning for periodontal instrumentation: A non-inferiority study | Oh, S. L.; Mishler, O.; Yang, J. S.; Barnes, C. | 2022 | Journal of dental education | Wrong outcomes; |
| Observation of classroom social communication: do children with fetal alcohol spectrum disorders spend their time differently than their typically developing peers? | Olswang, L. B.; Svensson, L.; Astley, S. | 2010 | Journal of Speech, Language & Hearing Research | Wrong outcomes; |
| Observed maternal strategies and children's health locus of control in low-income Mexican American families | Olvera, N.; Remy, R.; Power, T. G.; Bellamy, C.; Hays, J. | 2001 | Journal of family psychology : JFP : journal of the Division of Family Psychology of the American Psychological Association (Division 43) | Wrong outcomes; |
| Remote video audits of operating room practices sustainably improve patient safety processes and operating room throughput | Overdyk, F.; Dowling, O.; Di Capua, J. F.; Armellino, D.; Iammatteo, J.; Mercieca, R. | 2014 | Anesthesia and analgesia | Wrong outcomes; |
| Pilot randomized controlled trial of an attachment- and trauma-focused intervention for kinship caregivers | Pasalich, D. S.; Moretti, M. M.; Hassall, A.; Curcio, A. | 2021 | Child Abuse and Neglect | Does not meet inclusion criteria ; |
| How do medical specialists value their own intercultural communication behaviour? A reflective practice study | Paternotte, E.; Scheele, F.; van Rossum, T. R.; Seeleman, M. C.; Scherpbier, A. J.; van Dulmen, A. M. | 2016 | BMC medical education | Wrong outcomes; |
| TOP-DOWN AND BOTTOM-UP: THE ROLE OF SOCIAL INFORMATION PROCESSING AND MINDFULNESS AS PREDICTORS IN MATERNAL-INFANT INTERACTION | Pickard, J. A.; Townsend, M. L.; Caputi, P.; Grenyer, B. F. S. | 2018 | Infant Mental Health Journal | Wrong outcomes; |
| Interrater agreement for the schedule for affective disorders and schizophrenia epidemiological version for school-age children (K-SADS-E) | Polanczyk, V. G.; Eizirik, M.; Aranovich, V.; Denardin, D.; Da Silva, T. L.; Da Conceicao, V. T.; Pianca, T. G.; Rohde, L. A. | 2003 | Revista Brasileira de Psiquiatria | Wrong outcomes; |
| Distinguishing selective mutism and social anxiety in children: a multi-method study | Poole, Kristie L.; Cunningham, Charles E.; McHolm, Angela E.; Schmidt, Louis A. | 2021 | European Child & Adolescent Psychiatry | Wrong study design; |
| A simulation study to evaluate improvements in anesthesia work environment contamination after implementation of an infection prevention bundle | Porteous, G. H.; Bean, H. A.; Woodward, C. M.; Beecher, R. P.; Bernstein, J. R.; Wilkerson, S.; Porteous, I.; Hsiung, R. L. | 2018 | Anesthesia and analgesia | Wrong outcomes; |
| Development and validation of a tool for non-technical skills evaluation in robotic surgery-the ICARS system | Raison, N.; Wood, T.; Brunckhorst, O.; Abe, T.; Ross, T.; Challacombe, B.; Khan, M. S.; Novara, G.; Buffi, N.; Van Der Poel, H.; McIlhenny, C.; Dasgupta, P.; Ahmed, K. | 2017 | Surgical Endoscopy | Wrong outcomes; |
| The construction of power in family medicine bedside teaching: a video observation study | Rees, C. E.; Ajjawi, R.; Monrouxe, L. V. | 2013 | Medical education | Wrong outcomes; |
| Peer tutoring pilot program for the improvement of oral health behavior in underprivileged and immigrant children | Reinhardt, C. H.; Lopker, N.; Noack, M. J.; Klein, K.; Rosen, E. | 2009 | Pediatric Dentistry | Wrong outcomes; |
| Undergraduate medical education amid COVID-19: a qualitative analysis of enablers and barriers to acquiring competencies in distant learning using focus groups | Reinhart, A.; Malzkorn, B.; Doing, C.; Beyer, I.; Junger, J.; Bosse, H. M. | 2021 | Medical Education Online | Wrong outcomes; |
| Relationship Between Physiologically Measured Attention and Behavioral Task Engagement in Persons With Chronic Aphasia | Riley, Ellyn A.; Owora, Arthur | 2020 | Journal of Speech, Language & Hearing Research | Wrong study design; |
| The Impact of Rudeness on Medical Team Performance: A Randomized Trial | Riskin, Arieh; Erez, Amir; Foulk, Trevor A.; Kugelman, Amir; Gover, Ayala; Shoris, Irit; Riskin, Kinneret S.; Bamberger, Peter A. | 2015 | Pediatrics | Wrong outcomes; |
| Written evaluation is not a predictor for skills performance in an Advanced Cardiovascular Life Support course | Rodgers, D. L.; Bhanji, F.; McKee, B. R. | 2010 | Resuscitation | Wrong outcomes; |
| Does gender matter? addressing the question of gender superiority in resuscitation leadership | Rosenman, E. D.; Misisco, A.; Olenick, J.; Chipman, A. K.; Vrablik, M. C.; Brolliar, S. M.; Kalynych, C.; Grand, J.; Chao, G. T.; Kozlowski, S. W.; Fernandez, R. | 2020 | Circulation | Wrong outcomes; |
| A false sense of security: safety behaviors erode objective speech performance in individuals with social anxiety disorder | Rowa, Karen; Paulitzki, Jeffrey R.; Ierullo, Maria D.; Chiang, Brenda; Antony, Martin M.; McCabe, Randi E.; Moscovitch, David A. | 2015 | Behavior Therapy | Wrong outcomes; |
| A Video Analysis of Intra- and Interprofessional Leadership Behaviors Within "the Burns Suite": Identifying Key Leadership Models | Sadideen, H.; Weldon, S. M.; Saadeddin, M.; Loon, M.; Kneebone, R. | 2016 | Journal of surgical education | Wrong outcomes; |
| Enhancement of hand hygiene compliance among health care workers from a hemodialysis unit using video-monitoring feedback | Sanchez-Carrillo, L. A.; Rodriguez-Lopez, J. M.; Galarza-Delgado, D. A.; Baena-Trejo, L.; Padilla-Orozco, M.; Mendoza-Flores, L.; Camacho-Ortiz, A. | 2016 | American Journal of Infection Control | Incomplete dataset ; |
| Videotape review leads to rapid and sustained learning | Scherer, L. A.; Chang, M. C.; Meredith, J. W.; Battistella, F. D. | 2003 | American journal of surgery | Wrong outcomes; |
| Characterizing Novice Behavior Associated With Learning Ultrasound-Guided Peripheral Regional Anesthesia | Sites, B. D.; Spence, B. C.; Gallagher, J. D.; Wiley, C. W.; Bertrand, M. L.; Blike, G. T. | 2007 | Regional Anesthesia and Pain Medicine | Wrong outcomes; |
| Prevalence of Potentially Distracting Noncare Activities and Their Effects on Vigilance, Workload, and Nonroutine Events during Anesthesia Care | Slagle, J. M.; Porterfield, E. S.; Lorinc, A. N.; Afshartous, D.; Shotwell, M. S.; Weinger, M. B. | 2018 | Anesthesiology | Does not meet inclusion criteria ; |
| Decision-Making in Management of the Complex Trauma Patient: Changing the Mindset of the non-trauma Surgeon | Sonesson, L.; Boffard, K.; Lundberg, L.; Rydmark, M.; Karlgren, K. | 2018 | World Journal of Surgery | Wrong study design; |
| Video technology: use in nursing research | Spiers, J. A.; Costantino, M.; Faucett, J. | 2000 | AAOHN Journal | Wrong study design; |
| Does feedback matter? Practice-based learning for medical students after a multi-institutional clinical performance examination | Srinivasan, M.; Hauer, K. E.; Der-Martirosian, C.; Wilkes, M.; Gesundheit, N. | 2007 | Medical education | Wrong outcomes; |
| Will real-time feedback systems adversely affect colonoscopy practice? | Srinivasan, N.; Szewczynski, M. J.; Enders, F.; Tavanapong, W.; Oh, J.; Wong, J.; De Groen, P. C. | 2012 | Gastroenterology | Wrong outcomes; |
| Discovering communicative competencies in a nonspeaking child with autism | Stiegler, L. N. | 2007 | Language, Speech & Hearing Services in Schools | Wrong outcomes; |
| Psychophysiological responses to anger provocation among Asian Indian and white men | Suchday, S.; Larkin, K. T. | 2004 | International Journal of Behavioral Medicine | Wrong outcomes; |
| How surgical mentors teach: A classification of in vivo teaching behaviors part 2: Physical teaching guidance | Sutkin, G.; Littleton, E. B.; Kanter, S. L. | 2015 | Journal of surgical education | Wrong outcomes; |
| Patterns of premature newborns' sleep-wake states before and after nursing interventions on the night shift | Symanski, M. E.; Hayes, M. J.; Akilesh, M. K. | 2002 | Journal of obstetric, gynecologic, and neonatal nursing : JOGNN / NAACOG | Wrong setting; |
| Exploring patterns of error in acute care using framework analysis | Tallentire, V. R.; Smith, S. E.; Skinner, J.; Cameron, H. S. | 2015 | BMC medical education | Wrong outcomes; |
| Leadership training and quality improvement of interdisciplinary rounds in the ICU | Ten Have, E. C.; Tulleken, J. E. | 2013 | Critical Care | Wrong study design; |
| Assessment on Hand Hygiene Knowledge and Practices Among Pre-school Children in Klang Valley | Tengku Jamaluddin, T. Z. M.; Mohamed, N. A.; Mohd Rani, M. D.; Ismail, Z.; Ramli, S.; Faroque, H.; Abd Samad, F. N.; Ariffien, A. R.; Che Amir Farid, A. A. R.; Isahak, I. | 2020 | Global pediatric health | Wrong outcomes; |
| Audio-visual recording of patientâ€“GP consultations for research purposes: A literature review on recruiting rates and strategies | Themessl-Huber, Markus; Humphris, Gerry; Dowell, Jon; Macgillivray, Steve; Rushmer, Rosemary; Williams, Brian | 2008 | Patient education and counseling | Wrong study design; |
| Teaching teamwork during the Neonatal Resuscitation Program: A randomized trial | Thomas, E. J.; Taggart, B.; Crandell, S.; Lasky, R. E.; Williams, A. L.; Love, L. J.; Sexton, J. B.; Tyson, J. E.; Helmreich, R. L. | 2007 | Journal of Perinatology | Wrong outcomes; |
| Differences in gaze behaviour of expert and novice surgeons performing open inguinal hernia repair | Tien, T.; Pucher, P.; Sodergren, M.; Sriskandarajah, K.; Yang, G. Z.; Darzi, A. | 2014 | Surgical Endoscopy and Other Interventional Techniques | Wrong outcomes; |
| Impact of newborn screening & mediating factors on parentinfant relationships | Tluczek, A.; Clark, R.; McKechnie, A. C.; Brown, R. L. | 2012 | Pediatric pulmonology | Wrong outcomes; |
| Cognitive and affective characteristics of children with malformation syndrome | Tosi, B.; Maestro, S.; Marcheschi, M. | 1995 | Minerva pediatrica | Wrong outcomes; |
| Closing the gap: Using simulation to improve public health clinicians' affirming beliefs and behaviors with LGBT clients | Townsend-Chambers, Colette; Powers, Kelly; Coffman, Maren; Okoro, Florence; Robinson, Patrick A. | 2022 | Clinical Simulation in Nursing | Wrong study design; |
| Early young children's behavior during music and movement program | Tsapakidou, A. | 2001 | Journal of Human Movement Studies | Wrong outcomes; |
| Sharps handling practices among junior surgical residents-a video analysis | Tso, D.; Langer, M.; Blair, G.; Butterworth, S. | 2010 | Journal of Investigative Medicine | Wrong outcomes; |
| Impact of tailored feedback in assessment of communication skills for medical students | Uhm, S.; Lee, G. H.; Jin, J. K.; Bak, Y. I.; Jeoung, Y. O.; Kim, C. W. | 2015 | Medical Education Online | Wrong outcomes; |
| Transfer validity of laparoscopic knot-tying training on a VR simulator to a realistic environment: a randomized controlled trial | Verdaasdonk, E. G.; Dankelman, J.; Lange, J. F.; Stassen, L. P.; Verdaasdonk, E. G. G.; Dankelman, J.; Lange, J. F.; Stassen, L. P. S. | 2008 | Surgical Endoscopy | Wrong outcomes; |
| Verbal and non-verbal communication skills including empathy during history taking of undergraduate medical students | Vogel, D.; Meyer, M.; Harendza, S. | 2018 | BMC medical education | Wrong outcomes; |
| Mothering behavior and maternal role attainment during the postpartum period | Walker, L. O.; Crain, H.; Thompson, E. | 1986 | Nursing Research | Wrong outcomes; |
| The instructor's gaze guidance in video lectures improves learning | Wang, Hongyan; Pi, Zhongling; Hu, Weiping | 2019 | Journal of Computer Assisted Learning | Wrong study design; |
| Factors affecting feasibility and acceptability of a practice-based educational intervention to support evidence-based prescribing: A qualitative study | Watkins, C.; Timm, A.; Gooberman-Hill, R.; Harvey, I.; Haines, A.; Donovan, J. | 2004 | Family practice | Wrong study design; |
| Simulated Pediatric Resuscitation Use for Personal Protective Equipment Adherence Measurement and Training During the 2009 Influenza (H1N1) Pandemic | Watson, Christopher M.; Duval-Arnould, Jordan M.; McCrory, Michael C.; Froz, Stephan; Connors, Cheryl; Perl, Trish M.; Hunt, Elizabeth A. | 2011 | Joint Commission Journal on Quality & Patient Safety | Wrong outcomes; |
| Relational interaction in occupational therapy: Conversation analysis of positive feedback | Weiste, E. | 2017 | Scandinavian Journal of Occupational Therapy | Wrong outcomes; |
| Relational interaction in occupational therapy: Conversation analysis of positive feedback | Weiste, Elina | 2018 | Scandinavian Journal of Occupational Therapy | Wrong outcomes; |
| Applying evidence-based medicine in general practice: a video-stimulated interview study on workplace-based observation | Welink, Lisanne S.; Van Roy, Kaatje; Damoiseaux, Roger A. M. J.; Suijker, Hilde A.; Pype, Peter; de Groot, Esther; Bartelink, Marie-Louise E. L. | 2020 | BMC family practice | Wrong outcomes; |
| Comparing Performance Feedback and Video Self-Monitoring Within a BST Package to Train Pre-service Behavior Analysts to Conduct Preference Assessments | Weston, R.; Davis, T. N.; Radhakrishnan, S.; O'Guinn, N.; Rivera, G. | 2019 | Journal of Behavioral Education | Wrong study design; |
| Comparing Performance Feedback and Video Self-Monitoring Within a BST Package to Train Pre-service Behavior Analysts to Conduct Preference Assessments | Weston, R.; Davis, T. N.; Radhakrishnan, S.; O'Guinn, N.; Rivera, G. | 2020 | Journal of Behavioral Education | Wrong study design; |
| Teaching students behavior change skills: description and assessment of a new Motivational interviewing curriculum | White, L. L.; Gazewood, J. D.; Mounsey, A. L. | 2007 | Medical teacher | Wrong outcomes; |
| Getting Inside the Expert's Head: An Analysis ofÂ Physician Cognitive Processes During TraumaÂ Resuscitations | White, Matthew R.; Braund, Heather; Howes, Daniel; Egan, Rylan; Gegenfurtner, Andreas; van Merrienboer, Jeroen J. G.; Szulewski, Adam | 2018 | Annals of emergency medicine | Wrong study design; |
| Comparison of simulation-based interprofessional education and video-enhanced interprofessional education in improving the learning outcomes of medical and nursing students: A quasi-experimental study | Wu, Jen-Chieh; Chen, Hui-Wen; Chiu, Yu-Jui; Chen, Yi-Chun; Kang, Yi-No; Hsu, Yi-Ting; O'Donnell, John M.; Kuo, Shu-Yu | 2022 | Nurse Education Today | Wrong study design; |
| Determining influence, interaction and causality of contrast and sequence effects in objective structured clinical exams | Yeates, Peter; Moult, Alice; Cope, Natalie; McCray, Gareth; Fuller, Richard; McKinley, Robert | 2022 | Medical education | Does not meet inclusion criteria ; |
| Effect of non-technical skills teaching on performance-an evaluation of additional non-technical skills teaching in Resuscitation Council ALS course | Yeung, J.; Husselbee, N.; Davies, R.; Melody, T.; Lockey, A.; Gwinutt, C.; Bullock, I.; Gao, F.; Perkins, G. | 2015 | Resuscitation | Wrong outcomes; |
| Coaching Non-technical Skills Improves Surgical Residents' Performance in a Simulated Operating Room | Yule, S.; Parker, S. H.; Wilkinson, J.; McKinley, A.; MacDonald, J.; Neill, A.; McAdam, T. | 2015 | Journal of surgical education | Wrong outcomes; |
| Establishing Objective Measures of Clinical Competence in Undergraduate Medical Education Through Immersive Virtual Reality | Zackoff, Matthew W.; Young, Daniel; Sahay, Rashmi D.; Lin, Fei; Real, Francis J.; Guiot, Amy; Lehmann, Corinne; Klein, Melissa | 2021 | Academic pediatrics | Wrong outcomes; |
| Using video-based observation research methods in primary care health encounters to evaluate complex interactions | Asan, Onur; Montague, Enid | 2014 | Informatics in primary care | Wrong outcomes; |
| Merging video coaching and an anthropologic approach to understand health care provider behavior toward hand hygiene protocols | Boudjema, Sophia; Tarantini, ClÃ©ment; Peretti-Watel, Patrick; Brouqui, Philippe | 2017 | American Journal of Infection Control | Does not meet inclusion criteria ; |
| Effect of Videotape Playback and Teacher Comment on Anxiety During Subsequent Task Performance | Breen, Myles P.; Diehl, Roderick | 1970 |  | Wrong outcomes; |
| Impact of video-recording on patient outcome and data collection in out-of-hospital cardiac arrests | Dewolf, Philippe; Rutten, Boyd; Wauters, Lina; Van den Bempt, Senne; Uten, Thomas; Van Kerkhoven, Joke; Desruelles, Didier; Clarebout, Geraldine; Verelst, Sandra | 2021 | Resuscitation | Wrong outcomes; |
| Estimation of hand hygiene opportunities on an adult medical ward using 24-hour camera surveillance: validation of the HOW2 Benchmark Study | Diller, Thomas; Kelly, J. William; Blackhurst, Dawn; Steed, Connie; Boeker, Sue; McElveen, Danielle C. | 2014 | American Journal of Infection Control | Does not meet inclusion criteria ; |
| Differences in physicians' verbal and nonverbal communication with black and white patients at the end of life | Elliott, Andrea M.; Alexander, Stewart C.; Mescher, Craig A.; Mohan, Deepika; Barnato, Amber E. | 2016 | Journal of pain and symptom management | Wrong outcomes; |
| Study protocol for a framework analysis using video review to identify latent safety threats: trauma resuscitation using in situ simulation team training (TRUST) | Fan, Mark; Petrosoniak, Andrew; Pinkney, Sonia; Hicks, Christopher; White, Kari; Almeida, Ana Paula Siquiera Silva; Campbell, Douglas; McGowan, Melissa; Gray, Alice; Trbovich, Patricia | 2016 | BMJ open | Incomplete dataset; |
| Turn analysis and patient-centredness in paediatric otolaryngology surgical consultations | Forner, David; Ungar, Gilanders; Chorney, Jill; Meier, Jeremy; Hong, Paul | 2020 | Clinical Otolaryngology | Wrong outcomes; |
| Ethical and legal considerations in video recording neonatal resuscitations | Gelbart, Ben; Barfield, C.; Watkins, A. | 2009 | Journal of medical ethics | Wrong outcomes; |
| Social influence on handwashing with soap: results from a cluster randomized controlled trial in Bangladesh | Grover, Elise; Hossain, Mohammed Kamal; Uddin, Saker; Venkatesh, Mohini; Ram, Pavani K.; Dreibelbis, Robert | 2018 | The American journal of tropical medicine and hygiene | Wrong outcomes; |
| Hand hygiene compliance in the setting of trauma resuscitation | Haac, Bryce; Rock, Clare; Harris, Anthony D.; Pineles, Lisa; Stein, Deborah; Scalea, Thomas; Hu, Peter; Hagegeorge, George; Liang, Stephen Y.; Thom, Kerri A. | 2017 | Injury | Wrong outcomes; |
| CAMERA OBSERVATION SYSTEMS AND THE FEEDBACK ON THE HAND HYGIENE IMPROVEMENT STRATEGY AT THE NATIONAL CHILDRENâ€™S HOSPITAL 2015 | Huong, Dang Thi Thu; Le Thanh Hai, Le Kien Ngai; Van Ngoc, Phung | 2015 | system | Wrong outcomes; |
| Point-of-care assessment of medical trainee competence for independent clinical work | Kennedy, Tara J. T.; Regehr, Glenn; Baker, G. Ross; Lingard, Lorelei | 2008 | Academic Medicine | Wrong study design; |
| Reactivity beyond contamination. An integrative literature review of video studies in educational research | Lahn, Leif Christian; Klette, Kirsti | 2022 | International Journal of Research & Method in Education | Wrong study design; |
| Clinical Communication in Context: Applying Cognitive Role Theory in a Study of Physician Non-Verbals | Ledford, Christy J. W.; Canzona, Mollie Rose; Cafferty, Lauren A. | 2015 | Florida Communication Journal | Wrong outcomes; |
| The power of video recording: taking quality to the next level | Makary, Martin A. | 2013 | JAMA | Wrong study design; |
| Elbourne.(2013) Systematic review of the Hawthorne effect: New concepts are needed to study research participation effects | McCambridge, J.; Witton, J. | | Journal of clinical epidemiology | Wrong study design; |
| Systematic review of the Hawthorne effect: new concepts are needed to study research participation effects | McCambridge, Jim; Witton, John; Elbourne, Diana R. | 2014 | Journal of clinical epidemiology | Wrong study design; |
| Hand hygiene compliance monitoring: Do video-based technologies offer opportunities for the future? | McKay, Katherine J.; Shaban, Ramon Z.; Ferguson, Patricia | 2020 | Infection, Disease & Health | Wrong outcomes; |
| Hand hygiene compliance rates: fact or fiction? | McLaws, Mary-Louise; Kwok, Yen Lee Angela | 2018 | American Journal of Infection Control | Wrong study design; |
| Dynamic modeling of patient and physician eye gaze to understand the effects of electronic health records on doctorâ€“patient communication and attention | Montague, Enid; Asan, Onur | 2014 | International journal of medical informatics | Wrong outcomes; |
| Understanding the Hawthorne effect in wound researchâ€”A scoping review | Nguyen, Van N. B.; Miller, Charne; Sunderland, Janine; McGuiness, William | 2018 | International wound journal | Wrong study design; |
| Enriching medical traineesâ€™ learning through practice: a video reflexive ethnography study protocol | Noble, Christy; Billett, Stephen; Hilder, Joanne; Teodorczuk, Andrew; Ajjawi, Rola | 2019 | BMJ open | Wrong outcomes; |
| Obstetric competence and compliance with surgical hand antisepsis prior to elective and emergency surgical procedures: a closed-loop audit | Ooi, R.; Griffiths, A. | 2018 | Journal of Hospital Infection | Wrong study design; |
| Characterizing providersâ€™ immunization communication practices during health supervision visits with vaccine-hesitant parents: a pilot study | Opel, Douglas J.; Robinson, Jeffrey D.; Heritage, John; Korfiatis, Carolyn; Taylor, James A.; Mangione-Smith, Rita | 2012 | Vaccine | Wrong outcomes; |
| Using a self-regulated learning-enhanced video feedback educational intervention to improve junior doctor prescribing | Patel, Rakesh; Green, William; Shahzad, Muhammad Waseem; Church, Helen; Sandars, John | 2020 | Medical teacher | Wrong outcomes; |
| Video-based feedback of oral clinical presentations reduces the anxiety of ICU medical students: a multicentre, prospective, randomized study | Schmidt, Matthieu; Freund, Yonathan; Alves, Mickael; Monsel, Antoine; Labbe, Vincent; Darnal, Elsa; Messika, Jonathan; Bokobza, Jerome; Similowski, Thomas; Duguet, Alexandre | 2014 | BMC medical education | Wrong outcomes; |
| Evaluation of a training programme to induct medical students in delivering public health talks | Tan, Ngiap Chuan; Mitesh, Shah; Koh, Yi Ling Eileen; Ang, Seng Bin; Chan, Hian Hui Vincent; How, Choon How; Tay, Ee Guan; Hwang, Siew Wai | 2017 | Singapore medical journal | Wrong outcomes; |
| The psychology of camera observation: how the camera affects human behavior | Trainor, Catherine | 2021 |  | Wrong study design; |
| Observation for assessment of clinician performance: a narrative review | Yanes, Arianna F.; McElroy, Lisa M.; Abecassis, Zachary A.; Holl, Jane; Woods, Donna; Ladner, Daniela P. | 2016 | BMJ quality & safety | Wrong study design; |
