# Supplementary material for: Evaluating the impact of video cameras on participant behaviour in research: a systematic review and meta-analysis
Source: Syst Rev. 2026 Jan 24;15:65. doi: 10.1186/s13643-025-03055-z (PMC12911182; doi:10.1186/s13643-025-03055-z)
Supplement: Supplementary file 4 — Supplementary Material 4: Appendix 4: Data extraction plan. [file 13643_2025_3055_MOESM4_ESM.pdf]

Covidence	Study ID	Title	Reviewer N	Study ID (F	Title2	Lead authc	Journal	Impact fac	Country in

Covidence #
Study ID
Title
Reviewer Name
Study ID (First Author Name, Year)
Title2
Lead author contact details
Journal
Impact factor
Country in which the study conducted
Study design
Aim(s)
Study description
Start date (MM YYYY)
End date (MM YYYY)
Camera description
Camera size
Population description
Total number of participants
Intervention
Comparitor
Baseline Population Characteristics:
Female sex Overtly filmed
Female sex Covertly filmed
Female sex Directly observed
Female sex Total
Median age Overtly filmed
Median age Covertly filmed
Median age Directly observed
Median age Total
Mean age Overtly filmed
Mean age Covertly filmed
Mean age Directly observed
Mean age Total
Level of education Overtly filmed
Level of education Covertly filmed
Level of education Directly observed
Level of education Total
Median income Overtly filmed
Median income Covertly filmed
Median income Directly observed

Median income Total
Overtly filmed
Covertly filmed
Directly observed
Total
Qualitative summary
Subjective reportings:
Noticed camera Overtly filmed
Noticed camera Covertly filmed
Noticed camera Directly observed
Noticed camera Not filmed
Noticed camera Total
Initially reported feeling uncomfortable due to camera and later became comforable Overtly filmed
Initially reported feeling uncomfortable due to camera and later became comforable Covertly filmed
Initially reported feeling uncomfortable due to camera and later became comforable Directly observed
Initially reported feeling uncomfortable due to camera and later became comforable Not filmed
Initially reported feeling uncomfortable due to camera and later became comforable Total
Initially reported feeling anxious because of camera and later became comfortable Overtly filmed
Initially reported feeling anxious because of camera and later became comfortable Covertly filmed
Initially reported feeling anxious because of camera and later became comfortable Directly observed
Initially reported feeling anxious because of camera and later became comfortable Not filmed
Initially reported feeling anxious because of camera and later became comfortable Total
Reported changing behaviour because of camera Overtly filmed
Reported changing behaviour because of camera Covertly filmed
Reported changing behaviour because of camera Directly observed
Reported changing behaviour because of camera Not filmed
Reported changing behaviour because of camera Total
Reported feeling uncomfortable due to camera Overtly filmed
Reported feeling uncomfortable due to camera Covertly filmed
Reported feeling uncomfortable due to camera Directly observed
Reported feeling uncomfortable due to camera Not filmed
Reported feeling uncomfortable due to camera Total
Reported feeling anxious because of camera Overtly filmed
Reported feeling anxious because of camera Covertly filmed
Reported feeling anxious because of camera Directly observed
Reported feeling anxious because of camera Not filmed
Reported feeling anxious because of camera Total
Reported no concerns about camera Overtly filmed
Reported no concerns about camera Covertly filmed
Reported no concerns about camera Directly observed
Reported no concerns about camera Not filmed
Reported no concerns about camera Total
Reported did not notice camera Overtly filmed
Reported did not notice camera Covertly filmed

Reported did not notice camera Directly observed
Reported did not notice camera Not filmed
Reported did not notice camera Total
Satisfaction with experience Overtly filmed
Satisfaction with experience Covertly filmed
Satisfaction with experience Directly observed
Satisfaction with experience Not filmed
Satisfaction with experience Total
Objective reportings:
Looked at camera Overtly filmed
Looked at camera Covertly filmed
Looked at camera Directly observed
Looked at camera Total
Talking about camera Overtly filmed
Talking about camera Covertly filmed
Talking about camera Directly observed
Talking about camera Total
Gesturing towards camera Overtly filmed
Gesturing towards camera Covertly filmed
Gesturing towards camera Directly observed
Gesturing towards camera Total
Obstructing camera Overtly filmed
Obstructing camera Covertly filmed
Obstructing camera Directly observed
Obstructing camera Total
Whispering Overtly filmed
Whispering Covertly filmed
Whispering Directly observed
Whispering Total
Covering face Overtly filmed
Covering face Covertly filmed
Covering face Directly observed
Covering face Total
Adjusting clothes Overtly filmed
Adjusting clothes Covertly filmed
Adjusting clothes Directly observed
Adjusting clothes Total
Fixing hair/grooming Overtly filmed
Fixing hair/grooming Covertly filmed
Fixing hair/grooming Directly observed
Fixing hair/grooming Total
Applying makeup Overtly filmed
Applying makeup Covertly filmed
Applying makeup Directly observed
