# Supplementary material for: Evaluating the impact of video cameras on participant behaviour in research: a systematic review and meta-analysis
Source: Syst Rev. 2026 Jan 24;15:65. doi: 10.1186/s13643-025-03055-z (PMC12911182; doi:10.1186/s13643-025-03055-z)
Supplement: Supplementary file 5 — Supplementary Material 5: Appendix 5: Qualitative summary. [file 13643_2025_3055_MOESM5_ESM.docx]

| **Study ID** | **Title** | **Setting** | **Study description** | **Population description** | **Intervention** | **Comparitor** | **Outcome** |  |
| --- | --- | --- | --- | --- | --- | --- | --- | --- |
| Herzmark 1995 | Reactions of patients to video recording of consultations in general practice | UK - Clinical | This study films a number of patients visiting their GP's and compares their reactions, satisfaction, and rapport against a cohort who was not filmed. | Patients visiting a general practitioner in the UK | Video recorded consultation | Standard consultation without video recording | There is no major impact of filming on patients reports of the consultation. There is no significant difference in satisfaction and rapport between cohorts who were filmed and those who weren't. |  |
| Kabiri, 2020 | The Hawthorne Effect in Eye-blinking: Awareness that Oneâ€™s Blinks are Being Counted Alters Blink Behavior | United States – Non clinical | Participants were asked to complete a series of tasks, all whilst being video recorded, initially uninformed about the purpose of the study - they were led to believe that EEG recording was the focus. They were then informed that their blink rate was being measured and asked to repeat these tasks. Blink rate was measured through minute-by-minute counts by human raters based on video recordings of the subjects. | Adult Males [Mean Age 27.5 Â± 6.72 years], Uncorrected and Normal Vision | Informed Condition: Awareness that blink rate is being recorded | Uninformed Condition | Spontaneous Eye Blink Rate [SEBR] initially increased in minutes 1-3 following Informed Condition, but had no statistical difference following these minutes.  No comment on effect of video recording. |  |
| Rex et al. 2010 | The Impact of Videorecording on the Quality of Colonoscopy Performance: A Pilot Study | United States - Clinical | Routine colonoscopies were video recorded, initially without and then with the practitioners being aware of the videorecording, having now been informed that they were being recorded "for possible quality review". These recordings were blindly assessed for inspection time and technique to determine whether knowledge of being recorded impacted performance of the procedure. | 7 Endoscopists [98 Patients [?] - 98 Procedures, assuming new patient for each] | Awareness of videorecording for quality assessment | Videorecording without participant awareness | Awareness of videorecording improved colonoscopy performance. |  |
| McKay et al. 2022 | Health care workers' experience of video based monitoring of hand hygiene behaviours: a qualitative study | Australia - Clinical | This is a qualitative study involving 5 participants who were participants in a filmed clinical simulation using video monitoring of hand hygiene compliance. | Registrars and ICU nurses | Video recording |  | This qualitative study identified fears of participants which were mainly related to data breaches and inappropriate use of video footage. 4/5 participants reported that they forgot the camera was there after the initial few moments in the scenario. Only one person reported that they changed their behaviour because of the camera. |  |
| Manojlvich et al. 2019 | Formative evaluation of the video reflexive ethnography method, as applied to the physicianâ€“nurse dyad | United States - Clinical | An observational study to determine the impact of video reflexive practices can increase communication between nurses and doctors. | Nurses and physicians working in a teaching hospital | Video recording of ward rounds | None | This study was designed to describe the impact of a video reflection on the doctor-nurse relationship. The study showed only one dyad (2 participants) report that they were aware of the camera. This was not overtly asked during the interview process. |  |
| MacMurchy et al. 2017 | Acceptability, feasibility, and cost of using video to evaluate alarm fatigue | United States – Clinical | A survey administered to families who participated in a study that involved video recording. | Families of children admitted to hospital requiring intensive monitoring. | NA | None | All participants reported no change in their behaviour and the behaviour of their clinicians as a result of the video recording. |  |
| Gidlow et al. 2020 | Quantitative examination of video-recorded NHS Health Checks: comparison of the use of QRISK2 versus JBS3 cardiovascular risk calculators | UK - Clinical | This study evaluates cardiovascular risk discussion and in the primary care setting. It compares the use of two calculators. Video related behaviours are a secondary commentary. | Doctors and patients in the NHS primary care setting | NA | NA | 4 doctors involved in the simulations reported forgetting about the camera. |  |
| Aujla et al. 2021 | Development of a video-observation method for examining doctors' clinical and interpersonal skills in a hospital outpatient clinic in Ibadan, Oyo State, Nigeria | Nigeria - Clinical | This is an observational study assessing physician quality using video recording. Camera related behaviours and attitudes were a secondary outcome. | Resident clinicians in an outpatient department | Nil | Nil | One third of physicians said being on camera felt unnatural initially but found it easy after. Four ignored the camera. Two forgot it was there. |  |
| Weingarten et al. 2000 | A comparison of videotape and audiotape assessment of patient-centredness in family physicians' consultations | Israel - Clinical | Participating doctors had 2 hours of consultations recorded, whilst working as locum tenens in primary care. The first 2 minutes of these recordings were assessed using the patient centredness scale of Henbest and Steward, firstly with audio only, then with video recording included, to determine the loss of information which occurs when utilising audio versus video recording for physician assessment [i.e. non-verbal cues, origin of sound]. No significant difference was identified. | Locum General Practitioners |  | Audio v Video Recording | No difference in performance between audio and video performance |  |
| Wagner et al. 2021 | Video-based reflection on neonatal interventions during COVID-19 using eye-tracking glasses: an observational study | Netherlands - Clinical | In this study, proceduralists wore modern eye-tracking glasses during neonatal procedures to obtain point-of-view recordings, which were later observed and discussed as an educational tool, then assessed by observers to determine the recording's efficacy as an educational tool for specialist training and review of neonatal interventions via a questionnaire. Additionally, the proceduralists provided questionnaire feedback about the usability of these glasses during their procedures. | 12 Proceduralists 10 Patients 88 Observers | Eye-Tracking Glasses + Point-of-View Recording | NA | Proceduralist's subjective experience during procedures with Wilcoxon signed-rank test [outlined in subjective reportings table] showed they did not feel that their performance was influenced by the knowledge that the video recording may be reviewed, it did not distract them during the procedure and that they forgot that they were wearing eye-tracking glasses during the procedure. |  |
| Tipping et al. 1995 | Using Faculty and Student Perceptions of Group Dynamics to Develop Recommendations for PBL Training | Canada - Non clinical | A qualitative observational study, which combines prospective questions, post-tutorial questionnaires and live and videotaped observations to analyse faculty and student understanding of effective group dynamics. Observational data and self-reported data were compared to highlight inaccuracy of self-reported reflections. | 27 Undergraduate Medical Students 3 Faculty Members | Direct Observation & Video Recording | Self-Reporting | There was a lack of congruence between participant's self-reported and observed behaviours. |  |
| Rea et al. 2020 | Perceptions of scheduled vs. unscheduled directly observed visits in an internal medicine residency outpatient clinic | United States – Non clinical | Eight peer focus group sessions with semi-structures interviews were conducted, and used to explore the internal medicine resident and core teaching faculty perceptions of the scheduled and unscheduled direct observation methods in the outpatient clinic. | 148 Residents 50 Core Teaching Faculty [28 participants included in focus groups] | Scheduling of Observation | Unscheduled Observation | Scheduled observation residents stated they behave  differently when they know they are being observed and they might change how they act.   All residents in the scheduled and unscheduled groups preferred remote camera observation com-pared to in-room observation. |  |
| Ram et al. 1999 | Assessment of general practitioners by video observation of communicative and medical performance in daily practice: issues of validity, reliability and feasibility | Netherlands - Clinical | GP's were filmed during consultation the surveyed about their experience. | General Practitioners in the south of the Netherlands | Video recording | Nil | 71% of GP's reported that the camera did not change their behaviour. |  |
| Pringle et al. 1990 | Does awareness of being video recorded affect doctors' consultation behaviour? | UK - Clinical | This study offers no evidence that video recording has an effect on objective measures of doctors' consultation behaviour, and it offers support for the use of video recording as a tool for teaching and research. | General Practitioner | Awareness of Video Recording | Unaware of Video Recording | This study offers no evidence that video recording has an effect on objective measures of doctors' consultation behaviour, and it offers support for the use of video recording as a tool for teaching and research. |  |
| Pickering et al. 2014 | Video Surveillance Captures Student Hand Hygiene Behavior, Reactivity to Observation, and Peer Influence in Kenyan Primary Schools | Kenya – Non clinical | Student hand cleaning behaviour was monitored with video surveillance and in-person structured observation, both simultaneously and separately, at four primary schools in urban Kenya over a study period of 8 weeks. | Schoolchildren in Kenya | Video Observation Only | Direct Observation | We found evidence of student reactivity to both video surveillance and in-person observation, in the form of higher hand cleaning rates at handwashing with soap intervention schools. Reactivity was not detected at sanitizer intervention schools; this may be explained by the fact that hand cleaning rates with sanitizer were over 80%, leaving less room for compliance rates to shift upwards. Reactivity to simultaneous video and in-person observation increased handwashing rates by similar magnitudes (28% and 22%, respectively). These data should be interpreted as the marginal impact of each observation method on behavior when the other method is also in effect, as reactivity to each method could be higher when compared to a period during which no observation is occurring. |  |
| Penner et al. 2007 | Camera-Related Behaviours during Video Recorded Medical Interactions | United States - Clinical | Interactions between 45 patients and 14 medical oncologists were video recorded and coded for camera-related behaviours over two studies. | STUDY 1: 20 oncology patients and 7 physicians at a large cancer centre in southeastern US. STUDY 2: 25 oncology patients and 7 physicians at a large cancer centre in midwestern US. |  |  | Patients appear to habituate to the cameras rapidly, with the majority of camera-related behaviours occurring early in the consultation. Absence of the physician increased camera-related behaviours, likely due to attentional demands. The older the patients were, the fewer camera-related behaviours they displayed. |  |
| Miyazaki 2013 | Increasing Visual Search Accuracy by Being Watched | Japan – Non clinical | A non randomised experimental study examining participants performance in a basic task when overtly filmed vs not filmed. | College students | Overt surveillance + awareness of surveillance | Covert surveillance + no awareness of surveillance | Participants who were filmed searched slower and more thoroughly in performing the task comparted with those who were not filmed. |  |
| Martin et al. 1984 | The reactions of patients to a video camera in the consulting room | UK – Clinical | An observational study investigating patient's reaction to video recording during their consultation with their GP. | Patients being filmed during their consultations | Video recording |  | The majority of patients had no issues with the camera. 95% of patients subjectively reported that the camera did not change their behaviour. |  |
| Gross et al. 1993 | What does the NCATS measure? | United States – Non clinical | Mothers and their children were filmed while performing two teaching tasks. They were asked about their comfort on camera as a secondary analysis. | Mothers and toddlers |  |  | 25% of participants reported feeling uncomfortable due to the camera |  |
| Groener et al. 2015 | Video-based on-ward supervision for final year medical students | Germany – Non clinical | This study evaluates the effectiveness of video based proctorship and feedback in final year medical education. | Final year medical students. |  |  | All students reported that they fully disagreed with the statement they were ashamed to be filmed. All students found it valuable. |  |
| Ehsani et al. 2017 | Teen drivers' awareness of vehicle instrumentation in naturalistic research | United States – Non clinical | This study observes camera related behaviour over a course of 18 months. Semi structured interview and survey are used to identify subjective attitudes towards camera awareness. Objective measurements like G force measurements and accidents were compared to identify if the perception of the camera objectively caused behavioural change (eg. cautious driving). | Participants younger than 17 who had received their driving license within 3 weeks of study enrolment |  |  | Awareness of the camera reduced over time for all participants based on subjective reporting. There were no objective measures to suggest camera awareness. |  |
| Diller et. al 2013 | Electronic hand hygiene monitoring for the WHO 5-moments method | United States - Clinical | This study compares an electronic monitoring (gold standard) of hand gel dispensers, against video recording and direct observation. | Clinical staff in a medical unit. | Camera recording | Electronic monitoring of dispensed alcohol rub and direct observation. | There was a 30% difference in hand hygiene compliance comparing video recording and direct observation. Video recording had similar rates to an electronic monitoring sensor mounted to the dispenser suggesting no Hawthorne effect. |  |
| Coates et al. 2004 | Brief or New: Professional Development of Fieldwork Students: Occupational Adaptation, Clinical Reasoning, and Client-Centeredness | United States – Non clinical | This longitudinal observational study ran over 8 weeks and filmed student-patient interactions. Camera related behaviours were a secondary analysis. Most students appeared hesitant and apprehensive in the early phases of recording but all were comfortable by the end. There are no further specific definitions for camera related behaviour. | Occupational Therapy students | NA | NA | In the initial recordings most participants showed some degree of anxiety but this was gone by the last few sessions in almost everyone. |  |
| Castanelli 2009 | Use of video-assisted feedback to teach communication skills to trainees in paediatric anaesthesia | Australia - Clinical | This study used video recording as a means to provide more detailed feedback to trainees. Camera related attitudes were asked during a follow up survey. | Paediatric anaesthesia trainees | NA | NA | Some trainees reported anxiety and self consciousness as a result of being recorded. |  |
| Campbell et al. 1995 | Videotaping of general practice consultations: effect on patient satisfaction | UK - Clinical | This study was designed to determine if there were adverse reactions or clinical implications to having a GP consultation recorded from the patient's perspective. The primary outcome measured was patient satisfaction. | Patients visiting a general practitioner | Video recorded consultation | No video recording during consultation | Video recording did not impact patient satisfaction. |  |
| Beam et al. 2014 | Method for investigating nursing behaviors related to isolation care | United States - Clinical | This observational studies evaluated PPE use during a simulated scenario. Camera related behaviours were a secondary outcome. | Nurses working minimum 20 hours/week | Nil | Nil | Multiple errors in donning and doffing of PPE were made despite participants knowingly being recorded. |  |
| Antal et al. 2015 | Audio-Video Recording of Health Care Encounters for Pediatric Chronic Conditions: Observational Reactivity and Its Correlates | United States - Clinical | This observational study has the primary aim of examining camera related behaviours in paediatric clinics. All videos and events were coded using a validated metric and proportions of camera related behaviour were compared with all other behaviours. | Children who have a chronic health condition, their parents, and treating clinicians. | NA | NA | Of all coded events on video review, camcorder awareness events only accounted for 0.59%. Most parents, children, and practitioners did not change their behaviour due to presence of the camera. |  |
| Alsarhi et al 2021 | Observing sensitivity in slums in Yemen: the veiled challenge | Yemen – Non clinical | An observational study using a video camera to record and assess maternal sensitivity. This study is set in slums in Yemen and utilises a camera to film mother-child interactions. Many of the participants have never seen a camera before. Camera related behaviours was a secondary endpoint. | Mothers and children living in slums in Yemen | NA | NA | 90% of subjects looked at the camera. 40% expressed insecurity about their performance. 32% talked about being filmed. May subjects were amazed or entertained by the presence of a camera. Some subjects whispered around the camera. |  |
