# Supplementary material for: Evaluating the impact of video cameras on participant behaviour in research: a systematic review and meta-analysis
Source: Syst Rev. 2026 Jan 24;15:65. doi: 10.1186/s13643-025-03055-z (PMC12911182; doi:10.1186/s13643-025-03055-z)
Supplement: Supplementary file 6 — Supplementary Material 6: Appendix 6: Risk of bias analysis. [file 13643_2025_3055_MOESM6_ESM.docx]

| **Publication** | **Low** | **Moderate** | **High** | **Very High** |
| --- | --- | --- | --- | --- |
| Alsarhi et al. 2021 |  |  |  | * |
| Antal et al. 2015 |  |  |  | * |
| Aujla et al. 2021 |  |  |  | * |
| Beam et al. 2014 |  |  | * |  |
| Campbell et al. 1995 |  |  |  | * |
| Castanelli 2010 |  |  |  | * |
| Coates et al. 2004 |  |  |  | * |
| Diller et al. 2013 |  |  |  | * |
| Ehansi et al. 2017 |  |  | * |  |
| Gidlow et al. 2020 |  |  | * |  |
| Groener et al. 2015 |  |  |  | * |
| Gross et al. 1993 |  |  |  | * |
| Herzmark 1985 |  |  | * |  |
| Kabriri et al. 2020 |  |  |  | * |
| MacMurphy et al. 2017 |  |  |  | * |
| Majojlovich et al. 2019 |  |  |  | * |
| Martin et al. 1984 |  |  |  | * |
| McKay et al. 2022 |  |  |  | * |
| Miyazaki 2013 |  |  |  | * |
| Penner et al. 2007 |  |  | * |  |
| Pickering et al. 2014 |  |  |  | * |
| Pringle et al. 1990 |  |  | * |  |
| Ram et al. 1999 |  |  | * |  |
| Rea et al. 2020 |  |  |  | * |
| Rex et al. 2010 |  |  | * |  |
| Tipping et al. 1995 |  |  |  | * |
| Wagner et al. 2022 |  |  |  | * |
| Weingarten et al. 2001 |  |  |  | * |

**Risk of Bias Analysis**
