# Supplementary material for: Evaluating the impact of video cameras on participant behaviour in research: a systematic review and meta-analysis
Source: Syst Rev. 2026 Jan 24;15:65. doi: 10.1186/s13643-025-03055-z (PMC12911182; doi:10.1186/s13643-025-03055-z)
Supplement: Supplementary file 7 — Supplementary Material 7: Appendix 7: Studies included in meta-analysis. [file 13643_2025_3055_MOESM7_ESM.docx]

| \| **Studies included in final analysis** \| **Reported subjective behavioural change** \| **Reported participants noticing camera** \| **Reported concerns about camera** \| \| --- \| --- \| --- \| --- \| \| Alsarhi et al 2021 \|  \| X \|  \| \| Antal et al. 2015 \| X \| X \| X \| \| Aujla et al. 2021 \| X \| X \|  \| \| Beam et al. 2014 \|  \|  \| X \| \| Campbell et al. 1995 \|  \|  \|  \| \| Castanelli 2009 \|  \|  \|  \| \| Coates et al. 2004 \|  \|  \|  \| \| Diller et. al 2013 \|  \|  \|  \| \| Ehsani et al. 2017 \|  \|  \|  \| \| Gidlow et al. 2020 \| X \|  \|  \| \| Groener et al. 2015 \|  \|  \| X \| \| Gross et al. 1993 \| X \|  \|  \| \| Herzmark 1995 \| X \| X \| X \| \| Kabiri, 2020 \| X \|  \|  \| \| MacMurchy et al. 2017 \| X \|  \|  \| \| Manojlvich et al. 2019 \|  \| X \|  \| \| Martin et al. 1984 \| X \|  \| X \| \| McKay et al. 2022 \|  \| X \|  \| \| Miyazaki 2013 \|  \|  \|  \| \| Penner et al. 2007 \|  \|  \|  \| \| Pickering et al. 2014 \|  \|  \|  \| \| Pringle et al. 1990 \|  \|  \|  \| \| Ram et al. 1999 \| X \|  \| X \| \| Rea et al. 2020 \|  \|  \|  \| \| Rex et al. 2010 \|  \|  \|  \| \| Tipping et al. 1995 \|  \|  \|  \| \| Wagner et al. 2021 \|  \| X \|  \| \| Weingarten et al. 2000 \|  \|  \|  \| |  |  |
| --- | --- | --- | --- | --- | --- | --- | --- | --- | --- | --- | --- | --- | --- | --- | --- | --- | --- | --- | --- | --- | --- | --- | --- | --- | --- | --- | --- | --- | --- | --- | --- | --- | --- | --- | --- | --- | --- | --- | --- | --- | --- | --- | --- | --- | --- | --- | --- | --- | --- | --- | --- | --- | --- | --- | --- | --- | --- | --- | --- | --- | --- | --- | --- | --- | --- | --- | --- | --- | --- | --- | --- | --- | --- | --- | --- | --- | --- | --- | --- | --- | --- | --- | --- | --- | --- | --- | --- | --- | --- | --- | --- | --- | --- | --- | --- | --- | --- | --- | --- | --- | --- | --- | --- | --- | --- | --- | --- | --- | --- | --- | --- | --- | --- | --- | --- | --- | --- | --- |
|  |  |  |
|  |  |  |
|  |  |  |
|  |  |  |
|  |  |  |
|  |  |  |
|  |  |  |
|  |  |  |
|  |  |  |
|  |  |  |
|  |  |  |
|  |  |  |
|  |  |  |
|  |  |  |
|  |  |  |
|  |  |  |
|  |  |  |
|  |  |  |
|  |  |  |
|  |  |  |
|  |  |  |
|  |  |  |
|  |  |  |
|  |  |  |
|  |  |  |
|  |  |  |
|  |  |  |
